# Roman water management impacted the hydrological functioning of wetlands during drought periods

**DOI:** 10.1038/s41598-023-46010-5

**Published:** 2023-11-01

**Authors:** Fernando Gázquez-Sánchez, Francisco Jiménez-Espejo, Miguel Rodríguez-Rodríguez, Lucía Martegani, Claudia Voigt, Dolores Ruíz-Lara, Ana Moreno, Blas Valero-Garcés, Mario Morellón, Celia Martín-Puertas

**Affiliations:** 1 Department of Biology and Geology, Building CITE IIB, Universidad de Almería, Carretera de Sacramento s.n, La Cañada de San Urbano, 04120 Almería, Spain; 2 Andalusian Centre for the Monitoring and Assessment of Global Change (CAESCG), Building CITE V, University of Almería, 04120 Almería, Spain; 3 Instituto Andaluz de Ciencias de la Tierra (IACT), CSIC-UGR, 18100 Armilla, Spain; 4 Department of Physical, Chemical and Natural Systems, Pablo de Olavide University, 41013 Seville, Spain; 5 Oficina de Arqueología, Gerencia de Urbanismo de Córdoba, 14004 Córdoba, Spain; 6 Department of Environmental Processes and Global Change, Pyrenean Institute of Ecology (IPE) – CSIC, Campus de Aula Dei, Avda. Montañana, 1005, 50059 Zaragoza, Spain; 7 Department of Geodynamics, Stratigraphy and Paleontology, Faculty of Geological Sciences, Complutense University of Madrid, 28040 Madrid, Spain; 8 Department of Geography, Royal Holloway University of London, Egham, TW20 0EX UK

**Keywords:** Palaeoclimate, Limnology, Hydrology

## Abstract

During the Roman domain of the Iberian Peninsula (from 201 BCE to 460 CE) water management infrastructures were built to satisfy high water demand. However, whether the Roman activities affected the hydrological balance of Iberian wetlands remains unclear. Here, we investigate the paleo-hydrology of Lake Zóñar (southern Iberia) by using the stable isotopes (^16^O, ^17^O, ^18^O, ^1^H and ^2^H) of its gypsum (CaSO_4_·2H_2_O) sediments and reconstruct the isotopic composition of the lake water during Roman times. A period of recurrent lake low stand occurred between 2120 and 1890 cal. yr BP (ca. 170 BCE to 60 CE), coinciding with a relatively dry climate stage recorded by most regional paleoclimate archives. The stable isotopes and hydrochemistry of the lake water during gypsum precipitation are consistent with a shallow saline lake that evaporated under relative humidity ~ 10% lower than the present annual mean and at least 20% less rainfall amount. Our analytical and archeological findings support lake level lowering during the Roman period was probably caused by combined arid climate conditions and diversion of the inlets feeding the lake. Spring capturing was likely necessary to satisfy the high water demand of nearby Roman settlements, in the framework of a period of persistent droughts.

## Introduction

Mediterranean wetlands are biodiversity hotspots, greatly damaged over the past 2000 years due to human pressures and the current climate change^1^. Apart from their ecological values, lakes provide sedimentary records that are among the most useful paleoclimate archives in continental areas. However, interpretations of lacustrine records from recent millennia can be hampered by artificial perturbations caused by humans, including modification of the natural hydrological functioning of wetlands^2–6^.

The sedimentary sequences of lakes and wetlands in the Iberian Peninsula have been widely used to reconstruct the past climate in the Western Mediterranean^2–15^, among others. Such records are especially relevant to our understanding of environmental and ecosystem responses and resilience to anthropogenic impacts in the last millennia^2–4,8,10,11^. The first evidence for intense alterations of the landscape in Iberia came from the Copper/Bronze Age (4–3th millennia BCE)^12^, when forests suffered major retractions in southern Iberia. Nevertheless, it was during the Roman period (from 201 BCE to 460 CE) when anthropic influence became widespread and intensified. Metal mining, including Pb–Ag–Cu–Au exploitations, was one of the main economic activities of the Romans in the Iberian Peninsula^13^. Such activities left a deep imprint of environmental pollution, as revealed by multiple studies of lake and marine sediments^13–19^. Also, land use changes, deforestation and wildfire intensification during the Iberian Roman period have been documented by local and regional sedimentary sequences^6,7^.

The Ancient Romans are well-known for transporting and storing fresh-water resources by means of the construction of aqueducts, pipes, tunnels, canals, and bridges^20^. Remnants of these water management infrastructures are ubiquitous in the Iberian Peninsula^21^. In the Andalusia lowlands of the Guadalquivir River basin, southern Spain, water demand due to intensified farming may have increased from ~ 200 BCE, coinciding with the foundation of the Roman province of Hispania Ulterior. In particular, the Battle of Munda (45 BCE) triggered a major population redistribution in central Andalusia that intensified with the final climax of the Caesar’s civil war^22^. Latin authors reported the construction of irrigation infrastructures in rural areas during this period^23^. Whether Roman water management activities caused alteration of ecosystems in the southern Iberian Peninsula remains unclear to date.

Southern Iberian paleoclimate records indicate that climate during some stages of the Roman times was wet and warm^7,8,11,24–29^. Indeed, the period of Roman domain in the Iberian Peninsula coincided with relatively humid conditions. Hence, the so-called Iberian Roman Humid Period (IRHP; 2600–1600 cal. BP; 650 BCE to 350 CE) was defined for the first time from the partially varved sediments of Lake Zóñar^2^. This climate stage in the Iberian Peninsula can be considered as a regional expression of the “Roman Climatic Optimum” that was characterized by higher solar irradiance in the Northern Hemisphere, causing mild climatic conditions^30^. The Roman Empire experienced times of maximum expansion and great prosperity under such favorable climate^30^. However, several paleoclimate records show that this humid phase was punctuated by some short arid stages^2,7,8,14,18,19,24–30^, including a relatively dry episode that extended from ~ 100 BCE to ~ 100 CE^7,8,11,31^.

**Figure 1:**
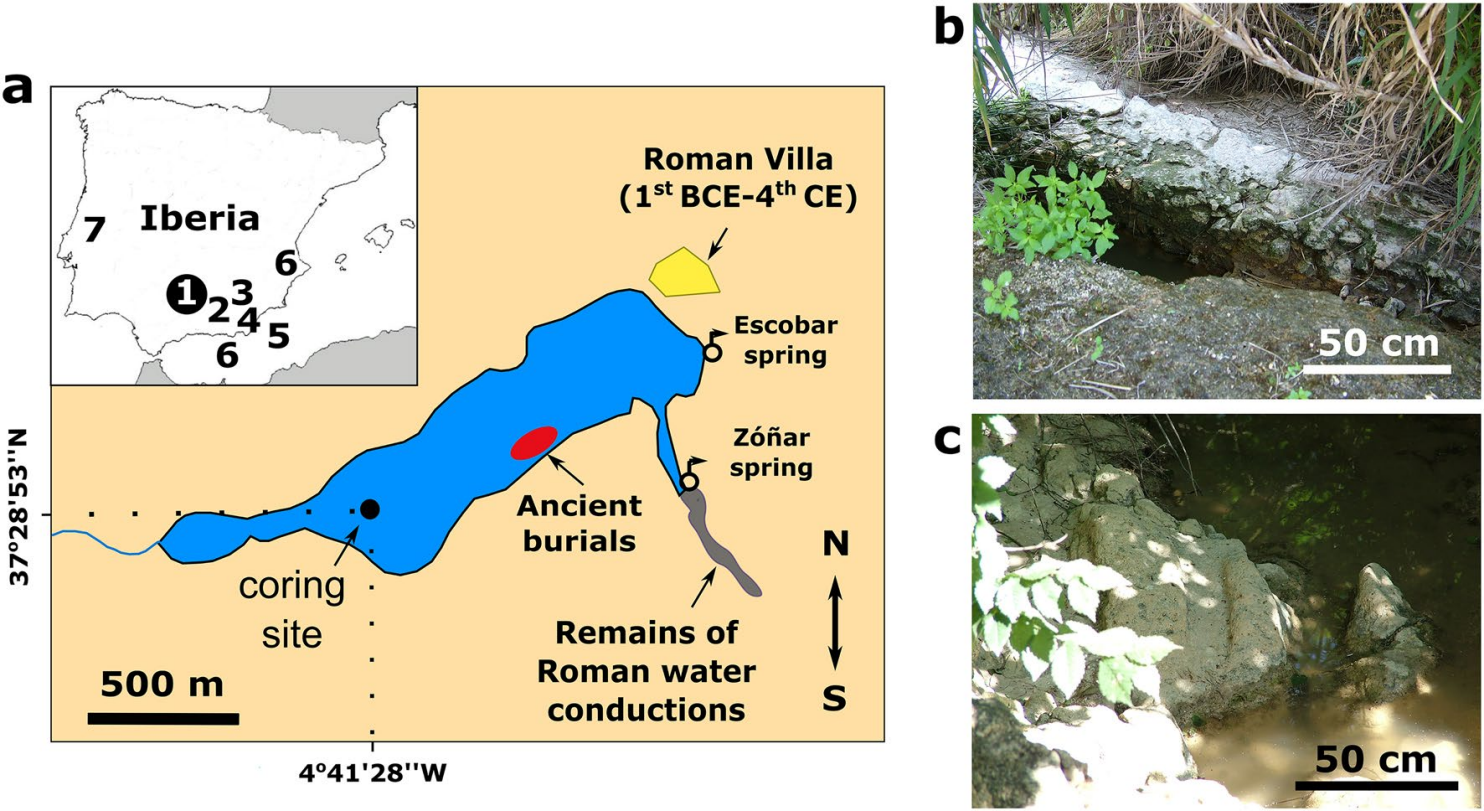
(**a**) Location of Lake Zóñar. The main paleoclimate records used for comparison in this study are indicated: (**1**) geochemical proxies of the Lake Zóñar sedimentary sequence^2,14,18,19^, (**2**) the Laguna de la Mula^7^ and (**3**) Borreguil de la Caldera lacustrine sequences^8^, (**4**) the Sima Blanca Cave gypsum stalactite^24^, (**5**) sediment cores from the Alboran Sea^14^, (**6**) pollen-based rainfall amount reconstructions from the southern Iberian Peninsula^11^ and (**7**) a carbonate speleothem record from Buraca Gloriosa Cave^25^. The coring site in Lake Zóñar is indicated in Fig. 1a, as well as the location of the main springs feeding the system. We also indicate the areas where remnants of Roman infrastructures have been found, including the vestiges of a rustic Roman *villae* (1st BCE century to fourth century CE) on the northwest shore of the lake [figure created by InkScape 0.92.4 (https://inkscape.org)]. Evidence of an ancient burial site, presumably of Roman age and usually under the water level, is also marked on the map, as well as remains of water conductions that channelized the Zóñar spring (Fig. 1b and 1c) (photographs taken by Dr. Dolores Ruíz-Lara).

In this study, we quantify the hydrological balance of Lake Zóñar (Córdoba Province, southern Spain) and atmospheric relative humidity in southern Iberian during some stages of the IRHP (Fig. 1). The sedimentary sequence of Lake Zóñar recorded several paleo-hydrological changes over the past 4000 years^2,14^. Gypsum (CaSO_4_·2H_2_O) is abundant in the sediment record from 200 BCE to 100 CE and its presence was initially interpreted as evidence for recurrent periods of droughts^2^. However, it remains unclear if the presumed low stands of the lake inferred from its mineralogy were induced by either climatic changes, human water management or a combination of both.

In order to shed light on this controversy, we analyze the stable isotopes of structurally-bonded gypsum hydration water (GHW) to reconstruct the triple oxygen and hydrogen stable isotope composition of the lake water at the time of gypsum precipitation during the IRHP. Then, we use an isotope mass balance model to quantitatively estimate past climate conditions (e.g., atmospheric relative humidity, evaporation-to-inflow ratio, etc.) at the time of gypsum precipitation^32–36^. In addition, we conduct hydrological modeling of the lake level under several scenarios, including climate aridification and artificial modification of the lake hydrological balance, in order to evaluate their impacts on the lake water level and hydrochemistry. We support our interpretations on archeological evidence of man-made constructions for water channelization in Lake Zóñar watershed. We ultimately aim to unearth the connection between Roman activities, climate and the hydrological evolution of Lake Zóñar during the IRHP.

## Modern lake climate setting and hydrogeology

Lake Zóñar (297 m a.s.l., 37^o^28′59″N; 4^o^41′20″W) is located in the lowlands of the Guadalquivir River Valley, southern Spain. It has a rectangular shape, a length of ca. 1300 m and an average width of ca. 300 m (Fig. 1 and Supplementary fig. S1). With a maximum depth of 16.5 m and a maximum volume of 4.4 hm^3^, it is the deepest and one of the very few permanent lakes in the Andalusian lowlands^37^. The flooded surface of the lake ranges from 35 to 55 ha. Its catchment is dominated by a hilly, smooth topography covering 10.1 km^2^, while the extension of the hydrogeological basin is slightly higher (ca. 12 km^2^).

**Figure 2:**
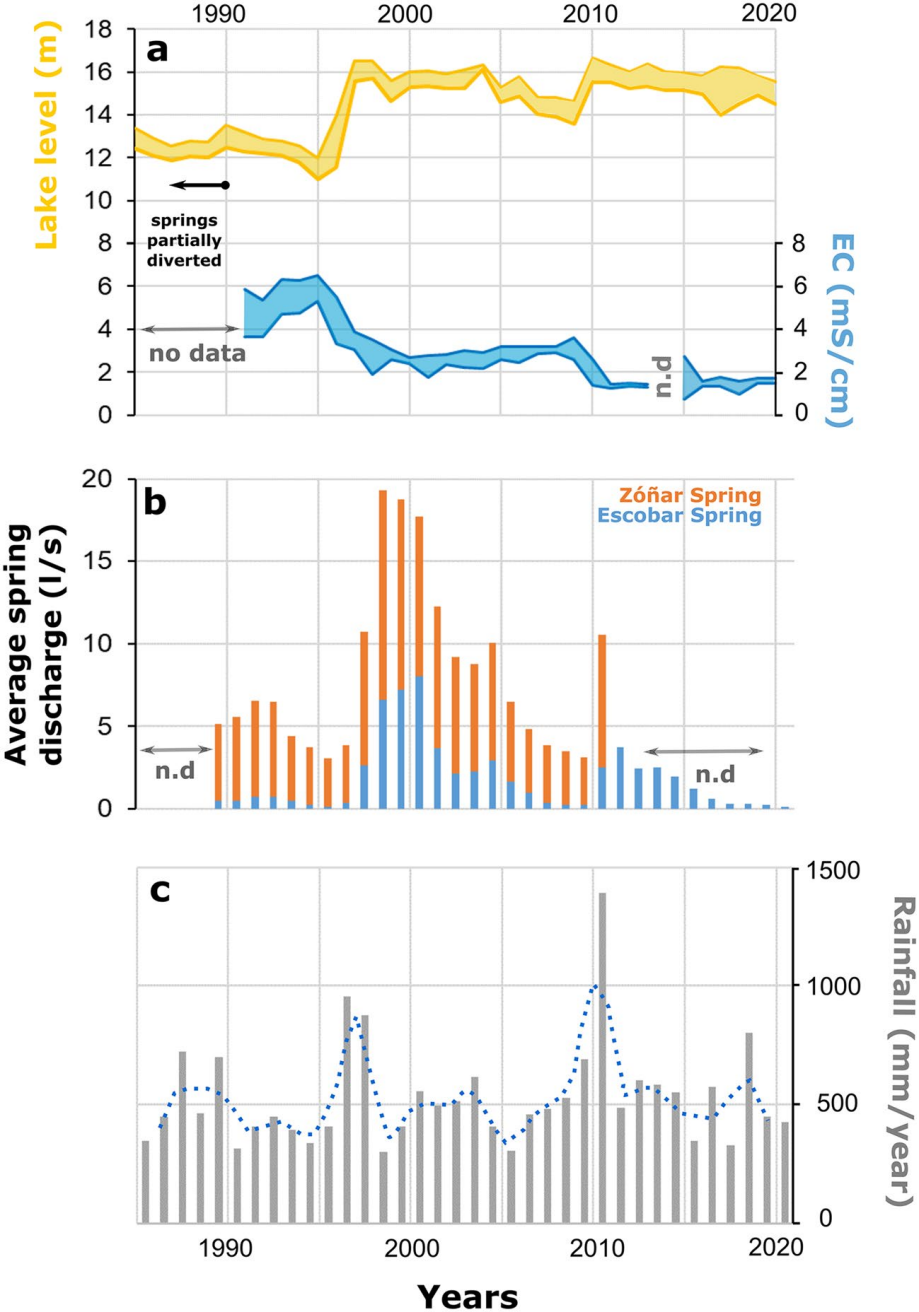
(**a**) Evolution of Lake Zóñar water level and water electrical conductivity (maximum and minimum annual values) from 1985 to 2020 (ref.^38^). No electrical conductivity data are available until 1992; (**b**) Discharge of Zóñar and Escobar springs to Lake Zóñar. No data are available until 1988 and from 2011 to 2020 for Zóñar spring; (**c**) Annual rainfall amount recorded by the meteorological station at Lake Zóñar (two year-moving average is displayed).

Lake water level, water chemistry and meteorological parameters have been monitored by the Natural Reserve of Lagunas del Sur de Córdoba over the past 35 years (1985–2021 period)^38^. Average precipitation in the Zóñar area is 533 mm/yr, the mean annual air temperature is 17.2 °C and mean annual relative humidity around 70% (Supplementary fig. S2). The mean potential evapotranspiration (PET) is about 880 mm/yr. The minimum depth of the lake was recorded in 1995 (11 m), coinciding with a period of persistent droughts (Fig. 2)^38^.

The aquifer under Lake Zóñar consists of relatively low permeability Upper Miocene sandstones and bioclastic limestones. In addition, permeable Quaternary sediments are hydraulically connected with the Upper Miocene sandstones^37^. Both Triassic clays with interbedded evaporites and Upper Miocene marls are low-permeable sediments that constitute the impervious basement of the aquifer. Zóñar and Escobar springs drain the permeable sediments, with an average flow of 7.5 l/s (1985–2021 period). These creeks flow just at the contact between the base of the upper sandstones and the lower Triassic clays and marls. Zóñar spring is a permanent water inlet to the lake, while Escobar spring dries out during persistent dry periods. There is an additional ephemeral creek (Eucaliptos spring) located to the southwestern lake margin, which flows only after exceptionally rainy episodes; thus, it does not contribute significantly to the lake water balance. The response of the Zóñar springs is delayed by approximately two years with respect to the local hydroclimate (Fig. 2). This is a consequence of the relatively low permeabilities of the materials in the sedimentary sequence (clays, marls, limestones and sandstones) that comprises the catchment of the springs, in addition to the low slope of the terrain^39^.

Previous hydrological studies on Lake Zóñar found that the modern lake system is fed by direct precipitation on the lake surface (25%), surface runoff generated over the watershed (45%) and groundwater discharge from the springs (30%)^39^. The mean total inflow to the lake is 1.04 hm^3^/yr, while the loss by evaporation is ~ 0.78 hm^3^/yr (ref.^39^). Therefore, the modern mean evaporation-to-inflow ratio is ~ 0.75. This implies that 25% of the inflow is lost by underground discharge to the aquifers and occasional surface outflow when the lake overflows after intense rainfall periods. Hence, the current system can be considered as a throughflow lake^39^ (Supplementary fig. S3).

## Results

### Stable isotopes of modern lake water and paleo-lake water reconstructed from gypsum hydration water

**Figure 3:**
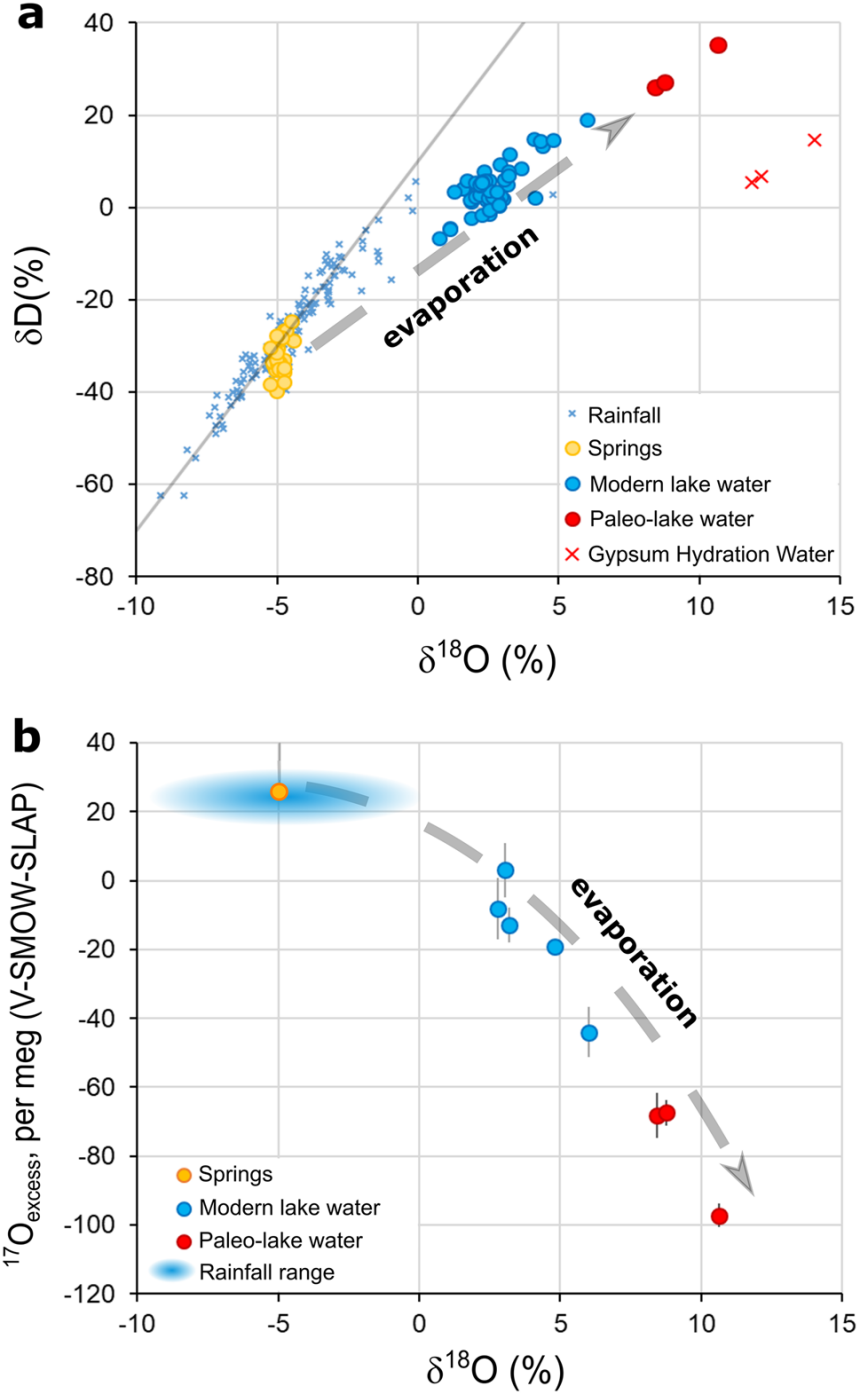
Isotopic composition (δ^18^O, δD and ^17^O_excess_) of Lake Zóñar water during periods of gypsum precipitation (between 2120 and 1890 cal. yr BP), reconstructed from GHW (after applying gypsum-water isotope fractionation factors)^32,33^, and modern lake waters)^2^ and this study. The δ^18^O and δD values of GHW are also displayed in panel (**a**). Overall, the results suggest that lake water was more evaporated at the stages of gypsum precipitation during the IRHP than at present.

The modern lake water ranges from 1.4 to 3.1‰ for δ^17^O, from 2.8 to 6.0‰ for δ^18^O, from 5.2 to 19.1‰ for δD, from − 8 to − 44 per meg for ^17^O_excess_ and from − 16.8 to − 28.9‰ for d-excess. The δ^17^O, δ^18^O and δD values increase from winter to summer and correlate negatively with d-excess and ^17^O_excess_ (Fig. 3 and Supplementary table S2). The δ^18^O and δD values of the lake water in 2020–2022 coincide with previously published data of the period 2001–2005 (ref.^2^). The mean isotopic composition of the springs feeding the lake in the 2001–2005 period was − 4.9 ± 0.2‰ for δ^18^O, − 32.2 ± 3.8‰ for δD, and 7.1 ± 3‰ for d-excess (ref.^2^). Similar values were measured in 2022 (one sample collected in July 2022; δ^18^O = − 4.9‰, δD = − 33.3‰ and d-excess = 6.3‰, with δ^17^O = -2.6‰ and ^17^O_excess_ = 22 per meg).

From 2120 to 1890 cal. yr BP, the isotope composition of GHW decreased from 7.3 to 6.2‰ for δ^17^O, from 14.1 to 11.9‰ for δ^18^O and from 14.6 to 5.5‰ for δD (Supplementary table S2 and Fig. 3). The oxygen and hydrogen isotope composition of the parent water from which the gypsum precipitated (hereafter referred to as paleo-lake water) is calculated from GHW using known fractionation factors^32,33^. Like the modern lake waters, primary isotope ratios show positive correlations, and correlate negatively to d-excess and ^17^O_excess_ (Fig. 3). In δD vs δ^18^O space, the paleo-lake waters follow an evaporation trend with a slope of 4.2, like that of modern lake waters (4.1) (Fig. 3). However, the paleo-lake waters show considerably higher δ^17^O (4.4–5.5‰), δ^18^O (8.4–10.6‰) and δD (26.0–35.3‰), and lower d-excess (− 41.4 to − 49.8‰) and ^17^O_excess_ (− 65 to − 95 per meg) values compared to modern lake waters.

### Archeological observations

Remnants of ceramic fragments and *opus tessellatum* (marble mosaic pieces) of a Roman *villa* (Roman farmhouse or country house) were found in the northeastern lake margin, occupied from the late first century BCE to ~ 4th/5th century CE, as estimated from the ceramic typology that is similar to that found in other nearby archaeological sites^40–42^, marble mosaic pieces and previous studies of Roman *villae* in this region^43^ (Supplementary fig. S8 and S9). The archaeological materials found in the Zóñar basin include red-gloss pottery (terra sigillata), tesserae (mosaic piece), fragments of marble used as pavement (opus sectile) and tegula fragments, among other buildings remnants.

In the southeastern margin of Lake Zóñar, close to the Zóñar spring, we found partially buried remains of Roman canals that were probably used to transport water from the creek to the Roman *villa* and nearby areas (Fig. 1). The construction typology is typically of Roman Epoch, including fragments of *opus signinum*, a form of concrete used commonly as building material with impervious characteristics, only during Ancient Roman Epoch. Opus signinum was common in baths, aqueducts, channels, cisterns and any building involving water^44^. This agrees with other major Roman hydraulic infrastructures that were completed in this area during the “Augustan period” (from 27 BCE to 14 CE), like the aqueduct of Ucubi (built during the late 1st BCE or early 1st century CE)^44^ and Valdepuentes aqueduct in Córdoba^45,46^.

## Discussion

**Figure 4:**
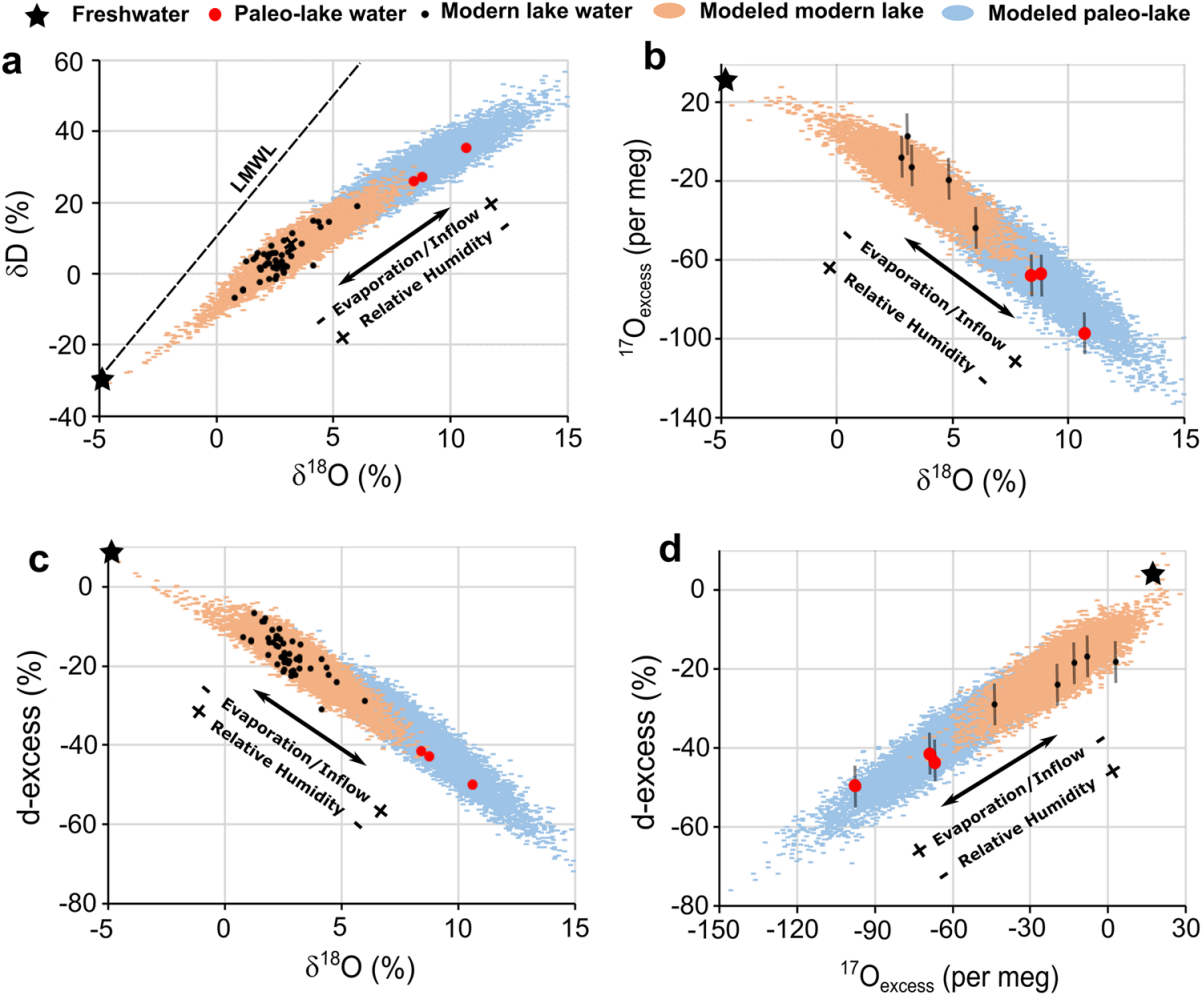
Isotope mass-balance modeling of the modern and paleo-water of Lake Zóñar. We use the methodological approach described in ref.^35^. Parametrization of the model used a suit of conservative hydrological parameters that cover the modern and potential conditions in the past (Supplementary table S5). The model satisfies simultaneously the δ^18^O, δD, d-excess and ^17^O_excess_ of the measured lake water. During the IRHP, the model implies slightly drier climate (relative humidity of ~ 55–60% compared to ~ 65–70% for modern conditions) and higher evaporation-to-inflow ratio (~ 1.4–1.6 compared to 0.6–1.0 for modern conditions) during the IRHP, suggesting more evaporated lake conditions and drier climate.

In Lake Zóñar, the presence of non-varved gypsum layers in sediment cores was initially interpreted as evidence for relatively dry conditions prevailing during some stages of the IRHP, from 2150 to 2050 cal. yr BP and from 1940 and 1870 cal. yr BP^2^. Our analyses of stable isotopes in gypsum hydration water reveal that the δ^17^O, δ^18^O and δD values in the paleo-lake water were higher and the d-excess and ^17^O_excess_ values were lower compared to modern lake water (Fig. 4). This strongly suggests that Lake Zóñar water was significantly more evaporated during the gypsum precipitation stages of the IRHP than at present. Indeed, higher ^17^O/^16^O, ^18^O/^16^O and ^2^H/^1^H ratios and lower d-excess and ^17^O_excess_ values in evaporated water are expected for lower evaporation-to-inflow ratio and lower atmospheric relative humidity^35,47,48^. Therefore, our results reveal that drier-than-present conditions prevailed during some stages of the IRHP in the setting of Lake Zóñar.

In order to quantify present and past hydroclimate conditions in the setting of Lake Zóñar, we used an isotope mass-balance model like ref.^35^ to reproduce the triple oxygen and hydrogen isotope composition of the modern and the paleo-lake water. We forced the model to use quasi-identical parameters for the modern and the past conditions, except for the relative humidity and the evaporation-to-inflow ratio (see supplementary material for parametrization). The best fit of the model to the modern lake water is observed for relative humidity of 70 ± 5% and evaporation-to-inflow ratio of 0.7 ± 0.2. This is similar to the results of the instrumental records and investigations on the current lake hydrology^38^. For the lake water during the IRHP, the best fit is obtained for relative humidity from 55 ± 2 to 59 ± 3‰ (at 2120 and 1890 cal. BP, respectively) and evaporation-to-inflow ratio from 1.6 ± 0.2 to 1.4 ± 0.2 (at 2120 and 1890 cal. BP, respectively) (Fig. 4 and Supplementary table S6).

The higher evaporation-to-inflow ratio (> 1) during the gypsum precipitation stages of the IRHP, between 2120 and 1890 cal. yr BP, reproduced by our model indicates desiccating conditions and significant water level lowering. This contrasts with the modern evaporation-to-inflow ratio (0.6–1.0) and maximum water levels of up to 16 m recorded over the past two decades (Fig. 2). Apart from a higher evaporation-to-inflow ratio, the model predicts relative humidity values slightly lower than the present annual mean (~ 10% lower) during the low stand stages of the IRHP. This indicates that the atmospheric relative humidity during the gypsum precipitation stages of the IRHP was lower than modern annual mean values and supports recurrent drier climate conditions between 2120 and 1890 cal. yr BP.

Today, the lake water is undersaturated in gypsum (SI_gyp_ < − 0.5) at any time of the year. The SI_gyp_ index of lake water correlated negatively with the lake water level in the period 1995–2021 (Supplementary fig. S5). Thus, during lake level high stand periods, the water is farther from gypsum saturation than during lake level low stand periods (R^2^ = 0.70; *p*-value = 10^–5^). There is a similar negative relationship between lake level and salinity (R^2^ = 0.92; *p*-value = 10^–7^) (Fig. 2 and Supplementary fig. S5). We conducted evaporation modeling using Phreeqc software in order to quantify the water loss by evaporation required to precipitate gypsum (Supplementary table S3). When starting from modern lake water composition (mean of 1991–2021), volume reduction of 84 ± 6% is required to reach gypsum saturation. According to the bathymetry of the lake, reduction to 16 ± 6% of the present volume corresponds to a lake level of 3.2 ± 0.5 m, ~ 12–10 m lower than the present mean value of 14.5 m. The estimated electrical conductivity of the lake water under such strong evaporative conditions is 34 ± 9 mS/cm, considerably higher than current electrical conductivity measured in the lake (~ 1–2 mS/cm), but similar to some saline playa-lakes of the Andalusian lowlands^39,49^.

**Figure 5:**
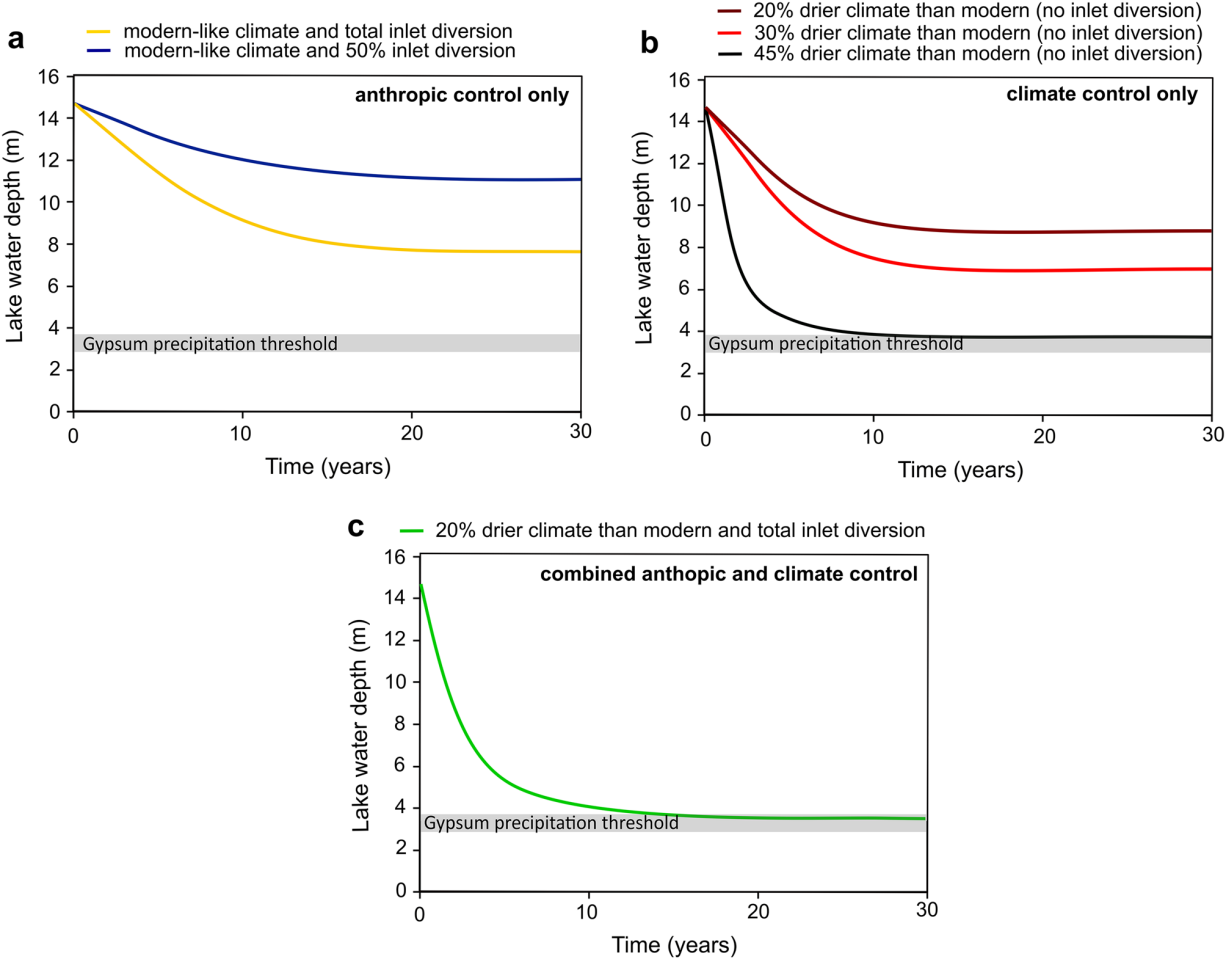
Lake Zóñar level modeling under different scenarios, including various degrees of spring diversion (**a**), natural climate aridification (**b**) and a combination of anthropic and natural climate causes (**c**). The lake level is forced to decrease to the threshold of gypsum saturation (lake level of 3.2 ± 0.5 m). This condition is achieved when assuming persistent dry climate conditions, 45% lower rainfall and spring discharge because of dry climate, or by combining dry climate conditions (e.g., 20% less rainfall amount and 20% more evapotranspiration than present annual mean) and total spring diversion.

To determine the potential causes (natural and/or anthropic) that led to lake water level lowering and gypsum precipitation between ~ 200 BCE and ~ 100 CE, we use a hydrological mass balance, to quantify the paleo-hydrology of Lake Zóñar under different scenarios. Under present-like environmental conditions, a partial diversion of the inputs by 50% produces lake level drop by 3.6 m compared to present (14.5 m), resulting in a level of 10.9 m above the lakebed (Fig. 5). Approximately 15 years are needed for the system to reach a new equilibrium state that depends on the surface-to-volume ratio of the lake, which controls the actual evaporation and the amount of direct precipitation received on the lake surface. The estimated impact of capturing 50% of the springs feeding the lake is consistent with the ~ 4 m lower-than-present lake level until 1990, when the Zóñar springs were partially diverted (34–67% of its volume) for urban supply. Note that after spring capturing ceased, the lake water level increased and stabilized around 14–16 m, from 1997 until 2020 (Fig. 2). The delayed response (ca. 7 years) of the lake level after spring diversion stopped was due to a succession of exceptionally dry years (< 380 mm/yr on average from 1990 to 1996) and low spring discharge (< 5 l/s) that maintained the lake level at its lowest values of the past 30 years.

In an additional run of our hydrological model, we found that a total diversion of the inlets feeding the lake under like-modern climate conditions results in lake level decline of 7.1 m compared to the modern mean value of 14.5 m, producing level stabilization at ~ 7.4 m above the lakebed. This is still significantly higher than 3.2 ± 0.5 m required to start gypsum precipitation (Fig. 5). This indicates that total diversion of the spring alone under like-modern or wetter climate cannot explain the lake level low stand between ~ 200 BCE and ~ 100 CE.

In a second scenario, we model the impact of natural climate aridification on the lake level, without springs diversion. The modeling results suggest that a consistent reduction by 20% in the water input to the lake (i.e., spring discharge, direct precipitation and runoff) during at least 20 years, e.g., caused by generally drier climate conditions as suggested by GHW modeling, results in lake level lowering of 5.6 m compared to the present mean value of 14.5 m (Fig. 5). The resulting water level of 8.6 m is also insufficient to reach the gypsum precipitation threshold at 3.2 ± 0.5 m. However, we found that a net reduction of the water input by 45%, including decrease in rainfall amount, consequent reduction in the flow of the spring and increased evaporation by 45%, during at least 10 years would result in a lake level of 3.6 m, ~ 11 m lower than the modern mean value of 14.5 m. This scenario is compatible (within uncertainties) with water evaporation and low stand needed for gypsum precipitation (3.2 ± 0.5 m).

**Figure 6:**
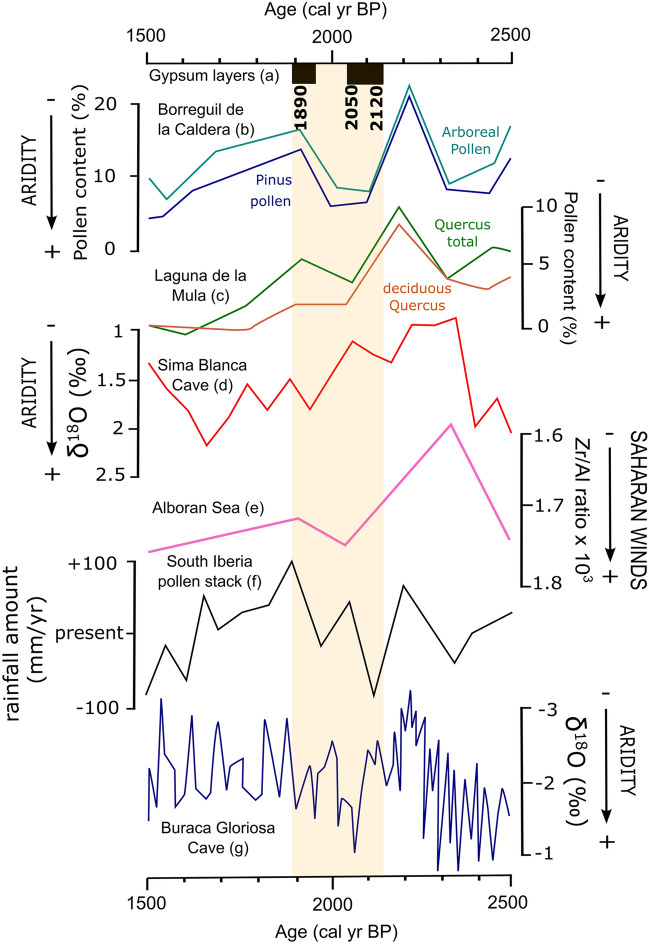
Comparison of Iberian paleoclimate records of the Iberian Roman Humid Period. Most records indicate alternation of dry and wet conditions during periods of gypsum precipitation in Lake Zóñar (**a**). This includes geochemical proxies from lakes in the Sierra Nevada area^7,8^ (**b, c**), a record of stable isotopes in gypsum hydration water from a gypsum stalactite from SE Spain^24^ (**d**), marine sediment from the Alboran Sea^14^ (**e**), stack pollen-based rainfall amount reconstruction from the Southern Iberian Peninsula^11^ (**f**) and carbonate speleothems from Buraca Gloriosa Cave in Portugal^25^ (**g**).

Most sedimentological studies in this region support the idea that the IRHP, despite being generally humid, was punctuated by decadal to centennial scale arid pulses^2,7,8,14,18,19,24–30^ (Fig. 6). Several lacustrine records from the nearby Sierra Nevada indicate a decrease in arboreal pollen, including *Pinus* and *Quercus* pollen, starting from ~ 2200 cal. yr BP. According to these records, a relative minimum of arboreal extension occurred between ~ 2100 and ~ 1900 cal. yr BP, suggesting a two centuries-long arid episode that preceded slightly wetter conditions at ~ 1900 cal. yr BP^7,8^. A similar pattern has been observed in the isotopic compositions of speleothems from Buraca Gloriosa in southern Portugal, with an excursion to relatively higher δ^18^O values at ~ 2050 cal. yr BP that is interpreted as a pulse of arid conditions^25^. An isotopic record from a gypsum speleothem in southeastern Iberia shows a gradual aridification trend from ~ 2300 to ~ 1600 cal. yr BP^24^, while an increase in the Zr/Al ratio in the sediments deposited in the Alboran Sea at ~ 2100 cal. yr BP was a consequence of stronger Saharan dust influxes because of drier regional conditions^14^. Also, pollen-based paleo-rainfall reconstructions in the southern Iberian Peninsula indicate that rainfall amount was ~ 100 mm/yr less compared to present during the driest stage of the IRHP (~ 2100 cal. yr BP; ref.^11^), coinciding with the episodes of gypsum precipitation in Lake Zóñar (Fig. 6). This is approximately 20% less annual precipitation amount than modern.

In a final run of our hydrological model, we combined anthropic (i.e., spring diversion) and climate controls (i.e., persistent droughts). We found that climate aridification by 20%, as suggested by ref.^11^, along with total spring capturing, results in lake level lowering to 3.5 m above the lakebed, similar (within uncertainties) to the lake level needed for gypsum precipitation (3.2 ± 0.5 m) obtained from our hydrochemical modeling. Therefore, the most likely cause of Lake Zóñar low stands during the IRHP that led to gypsum precipitation was a combination of dry climate (at least 20% less rainfall amount and 20% more evaporation) and artificial diversion of the springs, rather than a persistent 45% aridification alone.

Archaeological evidence supports that the setting of Lake Zóñar was inhabited and infrastructure for water channelization were built during Roman times. Roman *villae*, like the one found in the northeastern lake margin, were widespread in the Baetica province during that period^45,50,51^. The remnants of Roman channels found in the surroundings of the Zóñar spring strongly suggest that its flow was at least partially diverted during Roman times, likely to supply the Zóñar *villa* and nearby irrigation fields. Additional archaeological findings in the Lake Zóñar setting provide further evidence that the water level was considerably lower than modern in the past. The position of an undated burial site ~ 5 m below the modern water level implies that the lake level was permanently lower during some stages^46^. Based on previous Lake Zóñar level reconstructions during the last 3000 years^2,18,19^, lake low stands only occurred during Roman times, punctually during the Visigoth period (5th to 7th century CE)^2^ and in recent times. However, no evidence for Visigoth settlements in this area has been found to date. Therefore, it is possible that this ancient burial site was Roman in age.

## Conclusions

Combined analyses of triple oxygen and hydrogen isotopes in gypsum with hydrochemical and hydrological modeling are used here to untangle the role of climate and water management on the hydrology of Lake Zóñar during the Roman domain in the Iberian Peninsula. Our results reveal that lake level during some periods from ~ 200 BCE to ~ 100 CE was ~ 10–12 m lower and water salinity was considerably higher than at present, resulting in gypsum precipitation. The isotopic composition of the lake water at that time is consistent with a drier climate (10% lower relative humidity than modern annual mean). The hydrological conditions that prevailed to achieve such lake low stands required at least 20% less rainfall amount (ca. 100 mm/yr less) than at present, in combination with total or partial diversion of the springs feeding the lake. Archeological remnants in the surroundings of Lake Zóñar support the hypothesis of both spring water captured by the Romans and periods of lake low stands. Our study provides additional evidence for the high impact of the romanization on the ecosystems in Western Europe, and particularly in the Iberian Peninsula. Also, we conclude that past human interventions in the ecosystems need to be considered when interpreting lacustrine sedimentary sequences in terms of paleoclimate.

## Materials and methods

### Sampling of lake sediments

The 6-m-long core (ZON04-1B) investigated here was retrieved at 14 m water depth in the southwest part of Lake Zóñar (Fig. 1) in 2004, using a Kullenberg corer^2^. The sediments were stored at 4 °C in the Pyrenean Institute of Ecology (Zaragoza, Spain) until sampling of gypsum in 2019. The chronology of the ZON04-1B (6 m long) covers the last 4000 years and is based on 9 AMS ^14^C dates and ^137^Cs and ^210^Pb analyses in the upper section ^2,14,18,19^. The Zóñar age-depth model for the IRHP was later improved using a floating chronology based on varve counting linked to calendar time by radiocarbon dates^18,19^ (Supplementary fig. S6). We evaluate the impact of calibrating the ^14^C ages using the Intcal04 calibration curve as in ref.^2^ instead of the more recent Intcal20 calibration curve. We found that using either curve the calibrated ages are within uncertainties and the mean age difference is less than 10 yr (Supplementary table S1). Consequently, we use the age-depth model published previously in ref.^2^ that applies the Incal04 calibration curve.

The section of the core investigated (394–370 cm) includes a non-varved, gypsum-rich period with ages ranging from ~ 2140 to ~ 1800 cal. yr BP. Two gypsum-rich layers appear in ZON04-1B sediment core at depths from 394 to 385 cm (first gypsum precipitation stage; 2150–2050 cal. yr BP) and from 376 to 370 cm (second gypsum precipitation stage; 1940–1870 cal. yr BP). Randomly oriented prismatic gypsum crystals (20–100 μm) with sharp edges (Supplementary fig. S7) are interpreted as primarily formed underwater from a gypsum supersaturated solution on the lakebed^52^. Three gypsum samples (~ 300 mg each and ca. 1 cm resolution) taken at the depths of 391 cm (2120 cal. yr BP), 385 cm (2050 cal. yr BP), and 372 cm (1890 cal. BP) were analyzed for stable isotopes in GHW. These samples were dried in an oven at 40 °C over 24 h before being ground. Subsequently, the samples were dried for 24 h to remove any adsorbed moisture.

### Historical hydrochemical and meteorological data

We use the historical series of lake water level, water chemistry, meteorological parameters and spring volumes provided by the Natural Reserve of Lagunas del Sur de Córdoba^38^. Lake level was recorded monthly from 1985 to 2021 and water chemistry, including measurements of electrical conductivity (mS/cm), pH and temperature are available from 1991 to 2021. Major elements of monthly lake water from 1991 to 2021 were analyzed in the Laboratorio Agroalimentario de la Delegación Territorial de Córdoba de la Consejería de Agricultura, Pesca y Desarrollo Rural of the Andalusian Regional Government using Ionic chromatography. The meteorological station at Lake Zóñar provides air temperature, relative humidity and rainfall amount in hourly resolution from 1985 to 2021. Discharge rates and hydrochemical parameters of the Zóñar and Escobar spring were measured monthly from 1987 to 2010 and from 1987 to 2020, respectively.

### Stable isotopes of gypsum hydration water and lake waters

Following the method of ref.^53^, gypsum hydration water (GHW) was extracted by heating powdered gypsum (~ 300 mg) *in vacuo* and cryogenic trapping of water, using a bespoke offline system. The isotope composition (δ^17^O, δ^18^O, δD) of the extracted water was subsequently analyzed using a cavity ring-down spectroscopy (CRDS) analyzer (Picarro L2140i; Picarro, Inc., Santa Clara, California, USA) at the Laboratory of Stable Isotopes of University of Almería (Spain).

The δ^17^O and δ^18^O results were normalized to the V-SMOW-SLAP scale using four internal water standards analyzed before and after each set of 15 to 20 samples. These internal water standards (JRW, MILIQ, SPIT and ENR-15) were calibrated against V-SMOW and SLAP, using δ^17^O of 0.0‰ and -29.69865‰, respectively, and δ^18^O of 0.0‰ and -55.5‰, respectively. This standardization considers ^17^O_excess_ = 0 for both international standards as recommended by ref.^54^, where ^17^O_excess_ is defined as ln(δ^17^O/1000 + 1)–0.528 ln(δ^18^O/1000 + 1) (ref.^55^). The δD values were calibrated against V-SMOW, GISP and SLAP. All isotopic deviations are reported in parts per thousand (‰) relative to V-SMOW. The ^17^O-excess is given in per meg units (0.001‰). The d-excess is defined as δD-8*δ^18^O (ref.^56^). The reproducibility of the GHW method was ± 0.05‰ for δ^17^O, ± 0.1‰ for δ^18^O and ± 0.8‰ for δD, ± 1‰ for d-excess and ± 11 per meg for ^17^O-excess (1σ), based on the measurement of an internal gypsum standard (n = 3) analyzed alongside the samples. Analyses of modern lake waters (2020–2022) (n = 6) and Zóñar spring water (n = 1) (see Supplementary table S2 for sampling dates and results) used the same instrument and the same calibration, with similar analytical precision.

### Reconstruction of paleo-lake water isotope composition

The oxygen and hydrogen isotope compositions of the parent water from which gypsum formed is calculated from the measured isotope composition of GHW using known isotope fractionation factors (α_gypsum–water_) (ref.^32,33^). We use ^17^α_gypsum–water_ of 1.00180, ^18^α_gypsum–water_ of 1.00341 and ^2^α_gypsum–water_ of 0.980 (ref.^32,33^). Unlike stable isotopes of lacustrine carbonates, the oxygen and hydrogen isotope fractionations between the fluid and GHW are insignificantly affected by temperature and salinity during gypsum precipitation (for temperatures from 10 to 35 °C and salinities below 100 g/L)^32,33^. Importantly, recent investigations on GHW demonstrated that the primary lake water isotope composition can be preserved by lacustrine gypsum^34^.

### Isotope mass-balance modeling

We used a steady-state isotope-mass balance model (IMB) based on the Craig-Gordon evaporation model^57^, described in detail by ref.^35^. The model considers a suit of environmental parameters, including air and water surface temperature, atmospheric relative humidity, the degree of turbulences on the boundary layer (i.e., wind), water salinity, the evaporation-to-inflow ratio (E/I) of the lake, as well as the δ^17^O, δ^18^O, δD of the water input to the system and atmospheric water vapor. Monte Carlo simulations (R software^58^) are used to find the most probable solution to the model and evaluate uncertainties in relative humidity and the E/I ratio, resembling the approach used by ref.^35,36^. The error in each model solution is calculated relative to the mean and 1-sigma standard deviation (1SD) of each individual data point. Only those model solutions that fall within the 1SD are then selected. For model parametrization, we use conservative estimates based on the modern variability and their potential to change in the past (see Supplementary materials for model parametrization). In addition, model solutions for modern environmental conditions were compared to the measured isotopic composition of modern lake water samples collected in 2019–2022 (δ^17^O, δ^18^O and δD values) (Supplementary table S5).

### Hydrochemical modeling and paleo-hydrological balance

Hydrochemical data processing and modeling was made using Phreeqc v.3.7.1 (USGS, 2021). We calculated the gypsum saturation index (SI_gyp_) in modern lake waters and conducted evaporation modeling to assess the percentage of water loss required for the modern solution to reach gypsum saturation (SI_gyp_ = 0). The software provides the expected water salinity and conductivity at each evaporation stage. We use yearly data of ion concentrations, water temperature and pH from 1991 to 2021, including years of higher and lower lake levels (Supplementary table S3). The expected lake level at the stages of gypsum precipitation was determined using the lake water volume reduction needed for the solution to reach gypsum saturation and the Lake Zóñar volume-depth relationship from the bathymetric data obtained by ref.^39,59^ (Supplementary table S3 and Supplementary fig. S4).

We evaluate the impact of climate aridification and spring diversion on Lake Zóñar level by conducting hydrological balance modeling and lake level reconstructions, based on the hydrological model and the mass balance equation quoted “in section 5 of the supplementary material”. We use annual mean parameters (i.e., runoff, spring discharge, etc.) of the modern lake and the watershed calculated by ref.^39^, as estimated from meteorological parameters of the Lake Zóñar meteorological station and hydrological variables, such as direct measurements of the lake level and monthly spring discharge over the period 1985–1996 (12 years) (Supplementary table S4). The initial lake level was set to the mean value over the same period (14.5 m). We conducted two experiments to simulate the effect of artificial springs diversion on the lake level under like-today climate conditions, where the springs contribution to the lake is reduced by 50% and by 100% of its current mean flow, while the rest of parameters are held constant. Additionally, we simulated the effect of climate aridification by reducing the water inputs to the lake by 20%, 30% and 45%, and by increasing the evaporation output by the same amount, without modifying artificially the spring contributions. Finally, we explore the combined effect of climate aridification and anthropic spring diversion by reducing the rainfall/runoff input by 20%, by increasing the evaporation output by 20% and assuming total diversion of the springs.

### Archeological survey and chronology

Archaeological survey in the setting of Lake Zóñar consisted of surface recognition, mapping, and description of artifacts, using a similar approach to ref.^60,61^. To date, no scientific archeological excavations have been authorized in Zóñar basin. A field campaign was focused on the irrigation channel that channelized the Zóñar spring. The relative chronology of artifacts has been obtained from the description and classification of the existing ceramic material and channels, based on earlier archaeological investigations of this type of materials in other Roman settlements of central Andalusia^40–44^ (Supplementary fig. S8 and S9). We did not find any material suitable for radiocarbon dating or stratigraphic sequencing in our surface survey.

### Supplementary Information

Supplementary Information.

## Data Availability

All data generated or analyzed during this study are included in this published article and its supplementary information files. The historical series of lake water level, water chemistry, meteorological parameters and spring are accessible on request via e-mail to the Natural Reserve of Lagunas del Sur de Córdoba.

## References

[1] Perennou, Ch. *et al.* Evolution of wetlands in Mediterranean region. In *Water Resources in the Mediterranean Region* Eds: Zribi et al., M. 297–320 (2020).

[2] Martín-Puertas C (2008). Arid and humid phases in southern Spain during the last 4000 years: The Zóñar Lake record, Córdoba. Holocene.

[3] Gil-Romera (2010). Holocene fire activity and vegetation response in South-Eastern Iberia. Quat. Sci. Rev..

[4] Doyle (2022). Facies variability and depositional settings of Laguna Salada de Chiprana, an Iberian hypersaline lake. Sedimentology.

[5] Anderson RS, Jiménez-Moreno G, Carrión JS, Pérez-Martínez C (2011). Postglacial history of alpine vegetation, fire, and climate from Laguna de Río Seco, Sierra Nevada, southern Spain. Quat. Sci. Rev..

[6] Carrión JS (2003). Holocene vegetation dynamics, fire and grazing in the Sierra de Gador, southern Spain. Holocene.

[7] Jiménez-Moreno G (2013). Vegetation, fire, climate and human disturbance history in the southwestern Mediterranean area during the late Holocene. Quat. Res..

[8] Ramos-Román MJ (2016). Centennial-scale vegetation and north Atlantic oscillation changes during the late Holocene in the western Mediterranean. Quat. Sci. Rev..

[9] García-Alix (2022). Climatic control on the Holocene hydrology of a playa-lake system in the western Mediterranean. Catena.

[10] González-Sampériz P (2017). Environmental and climate change in the southern Central Pyrenees since the Last Glacial Maximum: A view from the lake records. Catena.

[11] Ilvonen (2022). Spatial and temporal patterns of Holocene precipitation change in the Iberian Peninsula. Boreas.

[12] Carrión JS (2007). Holocene environmental change in a montane region of southern Europe with a long history of human settlement. Quat. Sci. Rev..

[13] García-Alix A (2013). Anthropogenic impact and lead pollution throughout the Holocene in Southern Iberia. Sci. Total Environ..

[14] Martín-Puertas C (2010). Late Holocene climate variability in the southwestern Mediterranean region: An integrated marine and terrestrial geochemical approach. Clim. Past.

[15] López-Merino L, Cortizas AM, Lopez-Saez JA (2011). Human-induced changes on wetlands: A study case from NW Iberia. Quat. Sci. Rev..

[16] Ortiz JE (2021). Keys to discern the Phoenician, Punic and Roman mining in a typical coastal environment through the multivariate study of trace element distribution. Sci. Total Environ..

[17] Martínez-Cortizas A, Lopez-Merino L, Bindler R, Mighall T, Kylander M (2013). Atmospheric Pb pollution in N Iberia during the late Iron Age/Roman times reconstructed using the high-resolution record of La Molina mire (Asturias, Spain). J. Paleolimnol..

[18] Martín-Puertas C (2009). Geochemical processes in a Mediterranean Lake: A high resolution study of the last 4000 years in Zóñar Lake, southern Spain. J. Paleolimnol..

[19] Martín-Puertas C (2009). The Iberian-Roman Humid Period (2600–1600 cal. yr BP) in the Zóñar Lake varve record (Andalucía, Southern Spain). Quat. Res..

[20] Hodge, T. *Roman Aqueducts and Water Supply*, 2nd edn, Ed. Duckworth (2001).

[21] Fernández-Casado, C. *Acueductos romanos en España. Eds. Colegio de Ingenieros de Caminos, Canales y Puertos. Spain* (2008).

[22] González-Fernández, J. Colonización y municipalización cesariana en la Ulterior. In *Julio César y Corduba: Tiempo y espacio en la campaña de Munda (49–45 A.C.). Symposium*, 397–412 (University of Cordoba, 2005).

[23] Sánchez-López E (2008). Introducción a los acueductos romanos en Andalucía. Arqueología y Territorio.

[24] Gázquez F (2020). The potential of gypsum speleothems for paleoclimatology: Application to the Iberian Roman Humid Period. Sci. Rep..

[25] Thatcher DL (2020). Hydroclimate variability from western Iberia (Portugal) during the Holocene: Insights from a composite stalagmite isotope record. Holocene.

[26] Büntgen U (2011). 2500 Years of European climate variability and human susceptibility. Science.

[27] Bini M (2020). Hydrological changes during the Roman Climatic Optimum in northern Tuscany (Central Italy) as evidenced by speleothem records and archaeological data. J. Quat. Sci..

[28] Margaritelli G (2020). Persistent warm Mediterranean surface waters during the Roman period. Sci. Rep..

[29] McCormick M (2012). Climate change during and after the Roman Empire: Reconstructing the past from scientific and historical evidence. J. Interdiscip. Hist..

[30] Steinhilber F, Beer J, Fröhlich C (2009). Total solar irradiance during the Holocene. Geophys. Res. Lett..

[31] Drake BL (2017). Changes in North Atlantic oscillation drove population migrations and the collapse of the Western Roman Empire. Sci. Rep..

[32] Gázquez F, Evans NP, Hodell DA (2017). Precise and accurate isotope fractionation factors (α^17^O, α^18^O and αD) for water and CaSO_4_·2H_2_O (gypsum). Geochim. Cosmochim. Acta.

[33] Liu T (2018). Prediction of equilibrium isotopic fractionation of the gypsum/bassanite/water system using first-principles calculations. Geochim. Cosmochim. Acta.

[34] Gázquez F, Hodell DA (2022). Preservation and modification of the isotopic composition (^18^O/^16^O and ^2^H/^1^H) of structurally-bound hydration water of gypsum (CaSO_4_·2H_2_O) in aqueous solution. Geochim. Cosmochim. Acta.

[35] Gázquez F (2018). Triple oxygen and hydrogen isotopes of gypsum hydration water for quantitative paleo-humidity reconstruction. Earth Planet. Sci. Lett..

[36] Evans NP (2018). Quantification of drought during the collapse of the classic Maya civilization. Science.

[37] Dívar, J. *et al*. Mapa Geologico de España escala 1:50.000. IGME. Hoja 988 (1988).

[38] Natural Reserve of Lagunas del Sur de Córdoba. Memorias anuales de actividades y resultados. Junta de Andalucía (2021).

[39] Moral-Martos, F. *et al.* Definición del contexto hidrológico de humedales de la campiña andaluza central. Memoría técnica. Confederación hidrográfica del Guadalquivir. (2008). Junta de Andalucía public report https://www.chguadalquivir.es/documents/10182/52021/Humedales_Campina_Andaluza_I.pdf/62460c86-aa37-4630-91a9-b0a7e55b946a.

[40] Fernández Ochoa, C., Morillo Cerdán, A., Zarzalejos Prieto, M. (eds) Manual de cerámica romana II Cerámicas romanas de época altoimperial en Hispania: importación y producción. 1ª ed. Alcalá de Henares: Museo Arqueológico Regional; Madrid: Colegio Oficial de Doctores y Licenciados en Filosofía y Letras y en Ciencias, Sección de Arqueología, 536 p. Cursos de Formación Permanente para Arqueólogos (2015).

[41] Fernández Ochoa, C., Morillo Cerdán, A., Zarzalejos Prieto, M. (eds) Manual de cerámica romana IV. Producciones cerámicas de época medio-imperial y tardorromana. 1ª ed. Alcalá de Henares: Museo Arqueológico Regional; Madrid: Colegio Oficial de Doctores y Licenciados en Filosofía y Letras y en Ciencias, Sección de Arqueología. 672 p. Cursos de Formación Permanente para Arqueólogos (2019).

[42] Fernández Ochoa, C., Morillo Cerdán, A., Zarzalejos Prieto, M. (eds) Manual de cerámica romana III. Cerámicas romanas de época altoimperial III: cerámica común de mesa, cocina y almacenaje, imitaciones hispanas de series romanas, otras producciones. Alcalá de Henares: Museo Arqueológico Regional; Madrid: Colegio Oficial de Doctores y Licenciados en Filosofía y Letras y en Ciencias, Sección de Arqueología. 592 p. Cursos de Formación Permanente para Arqueólogos (2017).

[43] Hidalgo-Prieto, R. (Coord.) 2017. Las villas romanas en la Bética (Vol. I y II). Edit. Univ. de Sevilla. Páginas 1488 (2017).

[44] Roldan Gomez L (1992). El acueducto romano de Ucubi (Espejo, Córdoba). CuPAUAM.

[45] Ventura Villanueva, A. Los acueductos romanos de Córdoba y su rehabilitación Omeya. *Empúries* 53 (2002).

[46] Torres- Esquivias, J. A. Lagunas del sur de Córdoba. Eds. Diputación de Córdoba, Delegación de Medio Ambiente y Protección Civil (2004).

[47] Surma, J. *et al*. The evolution of ^17^O-excess in surface water of the arid environment during recharge and evaporation. *Sci. Rep.* **4972** (2018). 10.1038/s41598-018-23151-6 PMC5862851 29563523

[48] Voigt C (2021). Triple oxygen isotope systematics of evaporation and mixing processes in a dynamic desert lake system. Hydrol. Earth Syst. Sci..

[49] Rodríguez-Rodríguez M (2006). Estimation of ground-water exchange with semi-arid playa lakes (Antequera region, southern Spain). J. Arid Environ..

[50] Rodríguez-Neila JF (1993). Ciudad y territorio en la provincia romana de la Bética. Florentia Iliberritana: Revista de estudios de antigüedad clásica.

[51] Prieto, R. Las Villas Romanas de la Bética. Ed. Universidad de Sevilla. (2016).

[52] Cohen T (2022). Late quaternary climate change in Australia’s arid interior: Evidence from Kati Thanda-Lake Eyre. Quat. Sci. Rev..

[53] Gázquez F (2015). Simultaneous analysis of ^17^O/^16^O, ^18^O/^16^O and ^2^H/^1^H of gypsum hydration water by cavity ring-down laser spectroscopy. Rapid Commun. Mass Spectrom..

[54] Schoenemann SW, Schauer AJ, Steig EJ (2013). Measurement of SLAP and GISP δ^17^O and proposed VSMOW-SLAP normalization for ^17^O-excess. Rapid Commun. Mass Spectrom..

[55] Barkan E, Luz B (2005). High precision measurements of ^17^O/^16^O and ^18^O/^16^O ratios in H_2_O. Rapid Commun. Mass Spectrom..

[56] Dansgaard W (1964). Stable isotopes in precipitation. Tellus.

[57] Craig, H. & Gordon, L. I. Deuterium and oxygen 18 variations in the ocean and the marine atmosphere. In *Proc. Stable Isotopes in Oceanographic Studies and Paleotemperatures, Spoleto, Italy* (eds Tongiogi, E., V. Lishi e F.) 9–130 (1965)

[58] R Core Team R: A language and environment for statistical computing. R Foundation for Statistical Computing, Vienna, Austria. URL https://www.R-project.org/ (2022).

[59] Sánchez de la Orden, M., Fernández-Delgado, C. & Sánchez Polaina, F. Nuevos datos acerca de la morfometría y batimetría de la laguna de Zóñar (Aguilar de la Frontera, Córdoba). *Oxyura*.VI, Vol. 1, 73–77 (1992).

[60] Ruiz-Lara, M. D. Prospección arqueológica superficial en los términos municipales de Aguilar de la Frontera, Castro del Río, Montalbán, Montilla, La Rambla y Santaella Anuario arqueológico de Andalucía 1987, Vol. 2, 104–106 (1990).

[61] Ruiz-Lara MD, Murillo-Redondo JF (1992). Aproximación al Bronce Antiguo y Pleno en el sureste de la Campiña cordobesa: Los yacimientos del Cerro del Castillo de Aguilar y Zóñar. Anales de arqueología cordobesa.

