# Supplementary material for: Roman water management impacted the hydrological functioning of wetlands during drought periods
Source: Sci Rep. 2023 Nov 1;13:18815. doi: 10.1038/s41598-023-46010-5 (PMC10620414; doi:10.1038/s41598-023-46010-5)
Supplement: Supplementary file 1 — Supplementary Information. [file 41598_2023_46010_MOESM1_ESM.pdf]

Supplementary material to:

Roman water management impacted the hydrological functioning of wetlands during drought periods

Fernando Gázquez-Sánchez[1,2], Francisco Jiménez-Espejo[3], Miguel Rodríguez-Rodríguez[4], Lucía Martegani[1,2], Claudia Voigt[1], Dolores Ruíz-Lara[5], Ana Moreno[6], Blas Valero-Garcés[6], Mario Morellón[7] and Celia Martín-Puertas[8]

[1]Department of Biology and Geology, Building CITE IIB, Universidad de Almería, Carretera de Sacramento s.n, La Cañada de San Urbano, 04120 Almería, Spain

[2]Andalusian Centre for the Monitoring and Assessment of Global Change (CAESCG), Building CITE V, University of Almería, 04120 Almería, Spain

[3]Instituto Andaluz de Ciencias de la Tierra (IACT), CISC-UGR, Armilla, Spain

[4]Department of Physical, Chemical and Natural Systems, Pablo de Olavide University, Seville, Spain.

[5]Oficina de Arqueología. Gerencia de Urbanismo de Córdoba

[6]Department of Environmental Processes and Global Change, Pyrenean Institute of Ecology (IPE) – CSIC, Campus de Aula Dei, Avda. Montañana, 1005, E-50059, Zaragoza, Spain.

[7]Department of Geodynamics, Stratigraphy and Paleontology, Faculty of Geological Sciences, Complutense University of Madrid, 28040, Madrid, Spain.

[8] Department of Geography, Royal Holloway University of London, Egham TW20 0EX, United Kingdom.

1. **Modern Lake hydrogeology and geological setting**

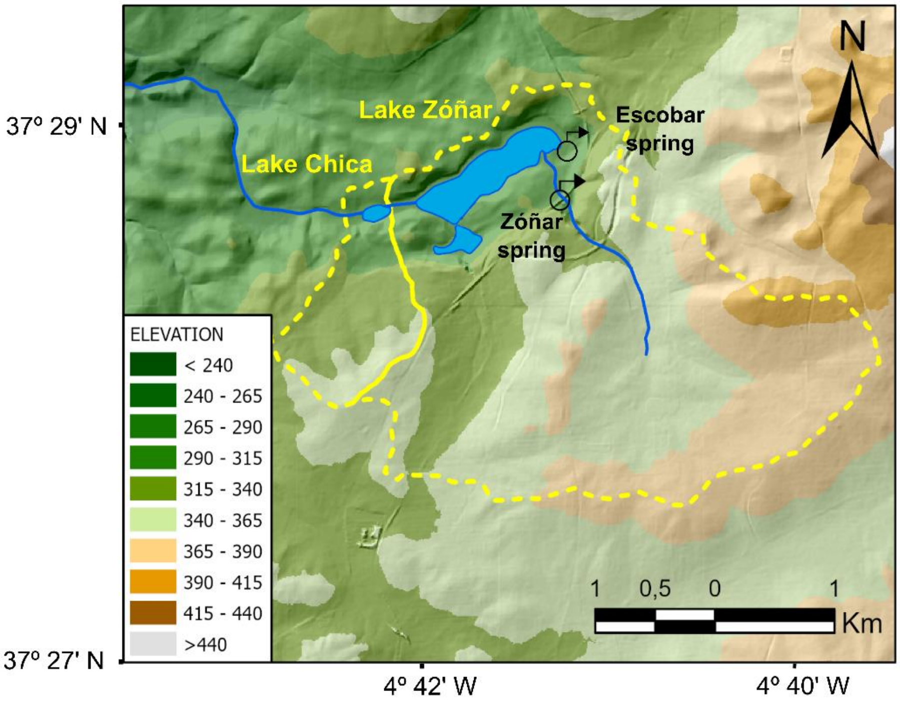

Supplementary figure S1. Topography, surface watershed and the main springs feeding Lake Zóñar. Figure created by InkScape 0.92.4 (https://inkscape.org).

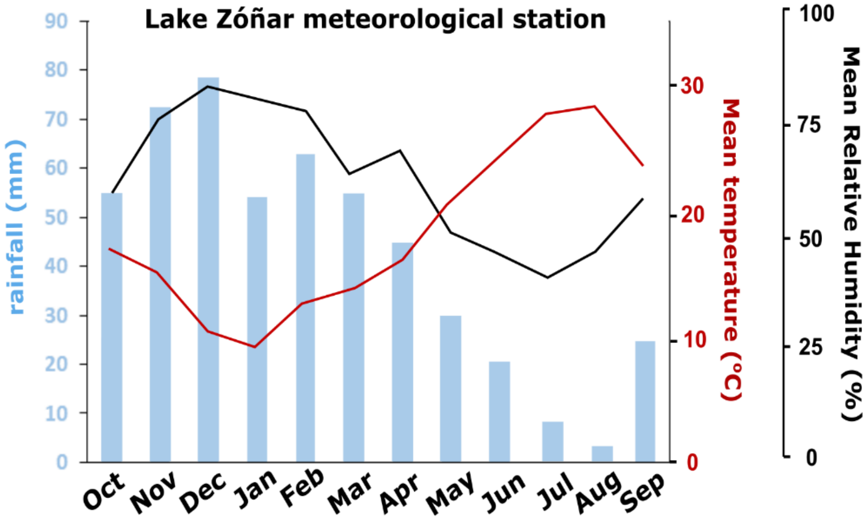

Supplementary figure S2. Mean hydrometeorological parameters measured by the Lake Zóñar meteorological station between 1982 and 2020.

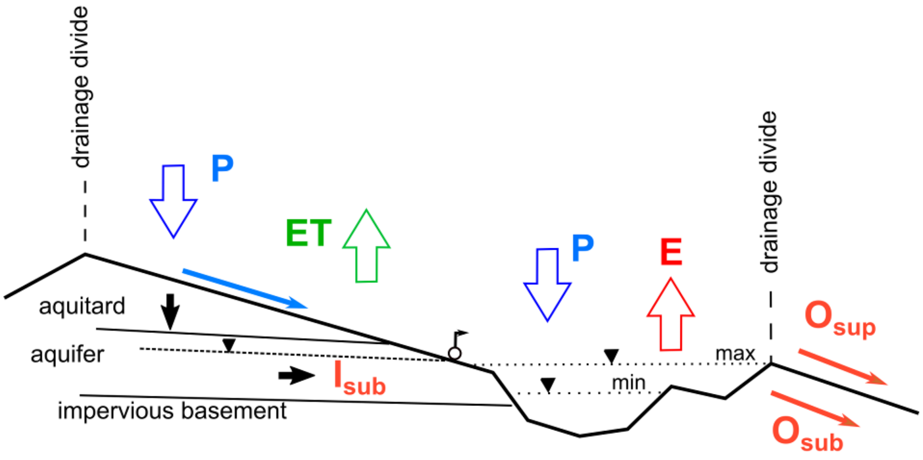

Supplementary figure S3. Schematic illustration of water inputs and outputs in Lake Zóñar considered in the hydrological model. P: precipitation; E: Lake water Evaporation; ET: Evapotranspiration; I_{sub}: groundwater inflow; O_{sup}: surface output; O_{sub}: subsurface output. Modified from Moral-Martos et al. (2008).

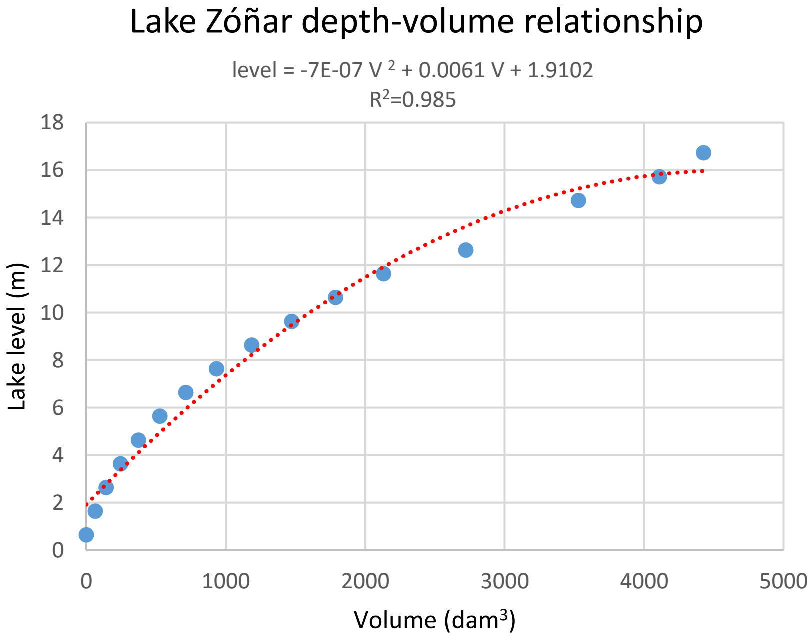

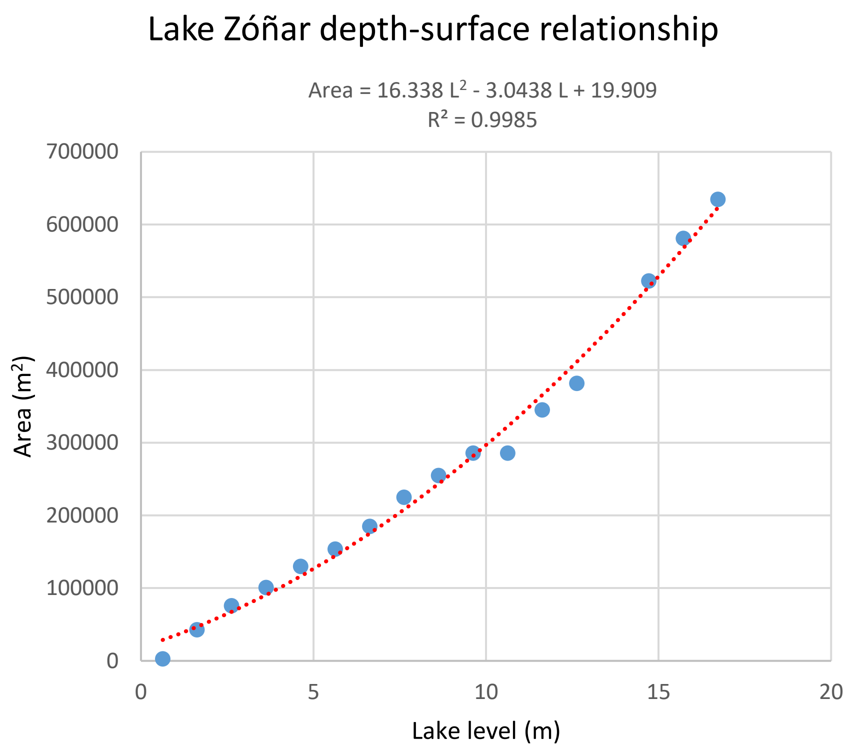

Supplementary figure S4. Depth-Volume relationship in Lake Zóñar (after Sánchez de la Orden et al. 1992).

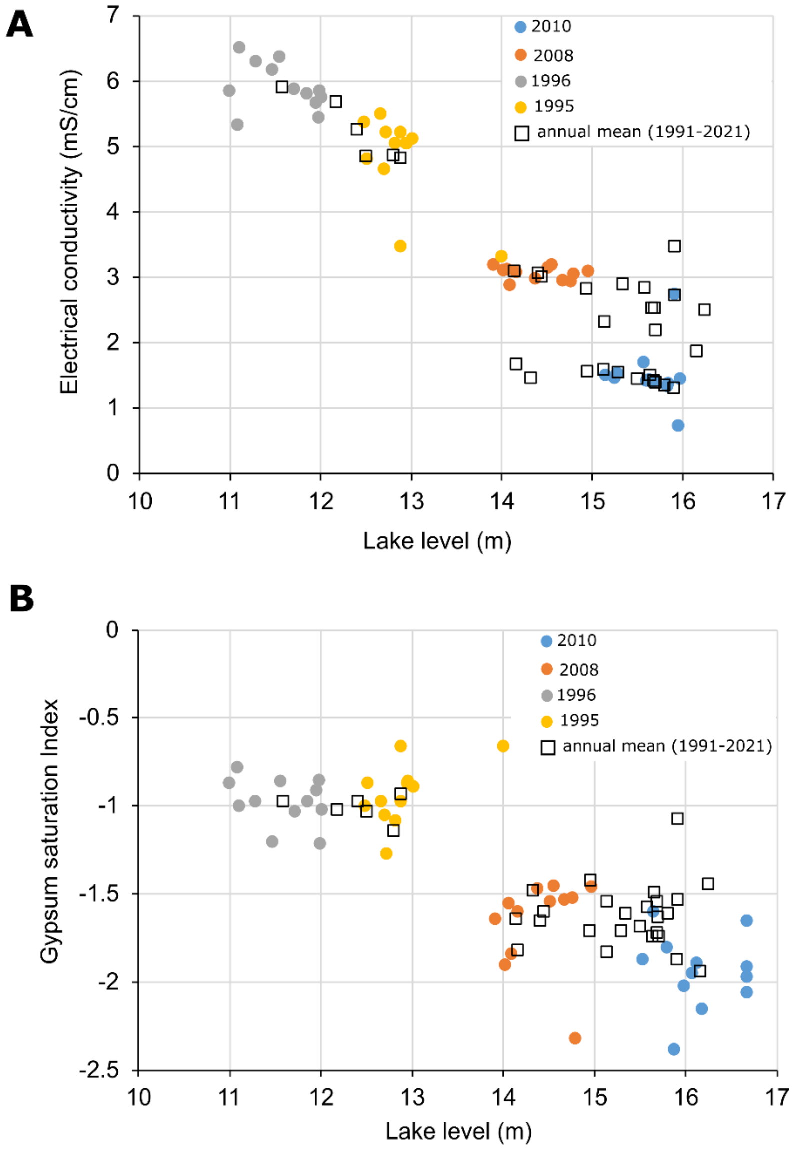

Supplementary figure S5. Cross plots of mean annual lake level vs water electrical conductivity and gypsum saturation index of water (SI_{gyp}) of Lake Zóñar from 1991 to 2021 (open squares). Also, monthly data are displayed (colored circles) for hydrological periods of high (e.g., 2010), low (e.g., 1995) and intermediate (e.g., 2008, 1996) lake level**.** The lake water is closer to gypsum saturation (SI_{gyp}=0) and higher electrical conductivity during low stand periods.

2. Sediment core and age model

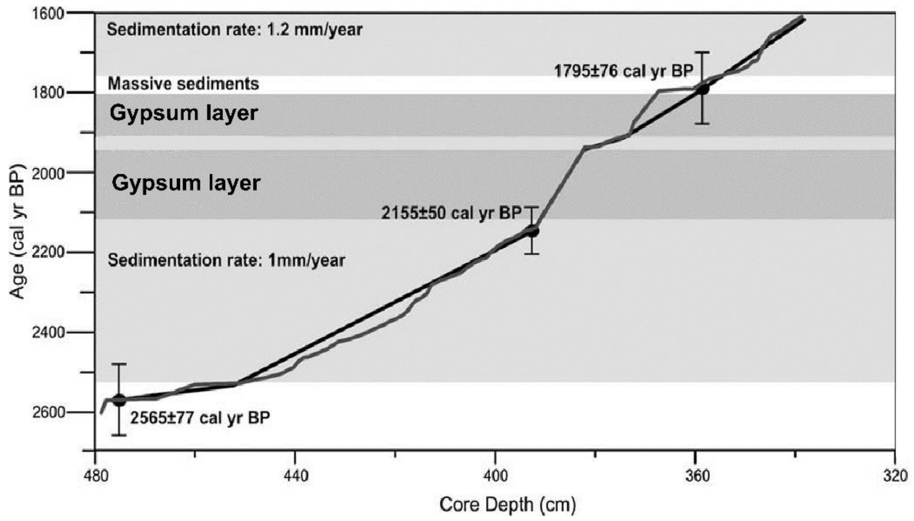

Supplementary Figure S6. Age-depth model of core ZON04-1B for the IRHP period in Lake Zoñar based on varve counting and AMS ^{14}C dates (modified from Martín-Puertas et al., 2009).

Lab sample ID	^{14}C age	Uncertainty (+/-)	cal. BP (Intcal 20) median	Uncertainty (+/-)	cal. BP (Intcal 04) median	Uncertainty +/-
Poz15971	330	30	390	80	390	72
AA47855	593	38	593	58	595	58
Poz18459	845	30	737	53	740	54
GRA-28167	825	40	732	59	735	61
Poz-16014	1350	30	1277	32	1275	38
Poz-18507	1865	30	1771	62	1795	73
Poz18460	2165	30	2116	66	2155	56
Poz18508	2525	30	2546	53	2565	77
GRA-28166	2595	40	2738	41	2740	43
GRA-30025	3145	40	3386	65	3385	65

Supplementary table S1. Chronological data of core ZON04-1B calibrated with the IntCal20 and IntCal04 curves.

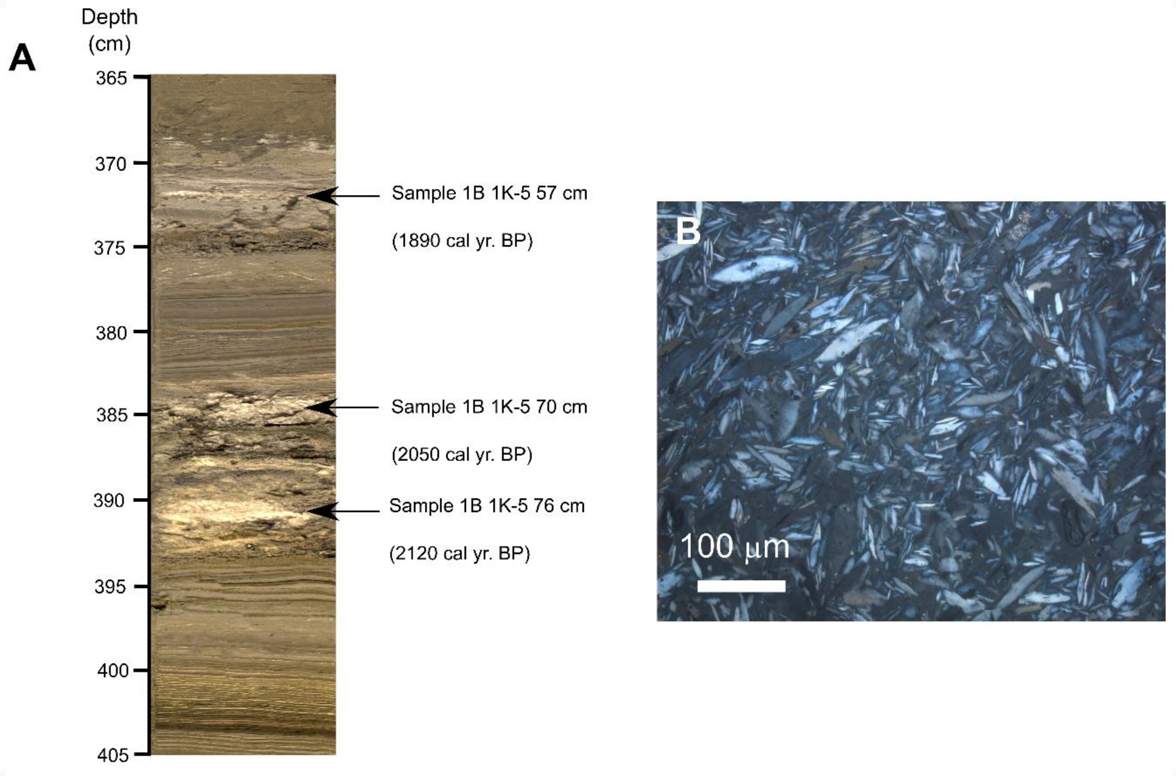

Supplementary figure S7. A. Sediment core ZON04-1B from depths of 405 to 365 cm, where gypsum layers appear. Sample ages were obtained from the age-depth model by Martín-Puertas et al. (2008), based on ^{14}C ages and varve counting; **B.** Microscope photography of gypsum crystals in the gypsum-rich layers of Lake Zóñar sedimentary record.

3. Stable isotope data

Supplementary table S2. Stable isotope composition of gypsum hydration water in Lake Zóñar and reconstructed isotope composition of the paleo-lake after applying known isotope fractionation factors (Gázquez et al., 2017). In addition, the stable isotope composition of modern lake water samples from 2020-2022 are presented. Additional lake water isotope data used in Fig. 3 of the main text were published previously by Martín-Puertas et al. (2009).

		GYPSUM HYDRATION WATER												PALEO-LAKE AND MODERN LAKE WATER											
**Sample**	**Age (cal yr BP)/date**	**$\delta^{17}O$**	**1SD**	**$\delta^{18}O$**	**1SD**	**δD**	**1SD**	**^{17}Oex**	**1SD**	**1SE**	**d-ex**	**1SD**	**1SE**	**$\delta^{17}O$**	**1SD**	**$\delta^{18}O$**	**1SD**	**δD**	**1SD**	**^{17}Oex**	**1SD**	**1SE**	**d-ex**	**1SD**	**1SE**
ZONAR 1B 1K-5 57 cm (gypsum)	1890	6.18	0.0	11.87	0.1	5.47	0.2	-65	16	7	-89.5	0.4	0.2	4.4	0.0	8.4	0.1	26.0	0.2	-66	16	7	-41.4	0.4	0.2
ZONAR 1B 1K-5 70 cm (gypsum)	2050	6.36	0.1	12.20	0.1	6.65	0.2	-64	9	4	-91.0	0.7	0.3	4.6	0.1	8.8	0.1	27.2	0.3	-65	9	4	-42.9	0.7	0.3
ZONAR 1B 1K-5 76 cm (gypsum)	2120	7.32	0.0	14.08	0.0	14.61	0.2	-94	8	3	-98.1	0.1	0.1	5.5	0.0	10.6	0.0	35.3	0.2	-95	8	3	-49.8	0.1	0.1
Lake Zóñar (modern) surface	28/07/2020	-	-	-	-	-	-	-	-	-	-	-	-	1.61	0.02	3.05	0.01	6.13	0.22	3	16	8	-18.3	0.3	0.2
Lake Zóñar (modern) bottom	28/07/2020	-	-	-	-	-	-	-	-	-	-	-	-	1.68	0.02	3.20	0.03	7.05	0.31	-13	14	5	-18.6	0.3	0.1
Lake Zóñar (modern) surface	19/03/2021	-	-	-	-	-	-	-	-	-	-	-	-	1.45	0.0	2.77	0.1	5.34	0.5	-8	24	9	-16.8	0.4	0.2
Lake Zóñar (modern) surface	30/06/2021	-	-	-	-	-	-	-	-	-	-	-	-	2.52	0.0	4.81	0.1	14.56	0.3	-19	6	2	-23.9	0.4	0.2
Lake Zóñar (modern) surface	02/07/2022	-	-	-	-	-	-	-	-	-	-	-	-	3.12	0.0	6.00	0.0	19.08	0.3	-44	18	7	-28.9	0.2	0.1
Zóñar spring	02/07/2022	-	-	-	-	-	-	-	-	-	-	-	-	-2.60	0.0	-4.96	0.0	-33.38	0.3	22	9	3	6.4	0.2	0.1

4. Calculations of saturation of gypsum saturation index, estimated water levels and salinities.

Supplementary table S3. Mean annual hydrochemical parameters and ionic concentrations in Lake Zóñar water from 1991 to 2021. Gypsum saturation index (SI_{gyp}) of water is calculated using PHREEQC software for each year. Concentration factors needed for the solution to reach gypsum saturation (SI_{gyp}=0) are calculated, as well as the estimated conductivity of the solution at the point of gypsum precipitation. The initial lake water volume for each year is estimated from the measured mean water level and the depth-volume relationship derived from bathymetric data (Supplementary fig. S4). The remaining volume is calculated by dividing the initial volume by the concentration factor.

YEAR	Level (m)	Conduct. (mS/cm)	pH	T (°C)	HCO_3^- (meq/l)	SO_4^{2-} (meq/l)	Cl^- (meq/l)	NO_2^- (meq/l)	Ca^{2+} (meq/l)	Mg^{2+} (meq/l)	K^+ (meq/l)	Na^+ (meq/l)	SI_{gyp}	Conc. fact	Initial volume (dam^3)	Remaining volume (dam^3)	Estimated Conduct. (mS/cm)	Estimated water level (m)
1991	12.8	4.9	8.5	16.5	4.0	4.9	41.4	2.5	3.5	7.3	0.5	55.1	-1.14	10.00	2658	266	42.4	3.5
1992	12.5	4.9	8.2	16.7	5.2	5.4	40.6	2.8	3.9	6.4	0.3	44.3	-1.03	7.93	2535	320	32.2	3.8
1993	12.4	5.3	8.3	16.3	4.7	5.8	44.8	2.7	4.3	7.1	0.5	42.7	-0.97	6.86	2495	364	29.5	4.0
1994	12.2	5.7	8.5	17.4	4.1	6.3	46.7	2.8	3.62	7.59	0.46	44.3	-1.02	7.91	2404	304	35.4	3.7
1995	11.6	5.9	8.1	16.5	5.3	6.7	48.5	4.1	4.10	8.97	0.46	46.3	-0.97	6.94	2175	313	32.8	3.8
1996	12.9	4.8	8.0	16.7	4.1	5.9	40.2	3.4	4.7	6.9	0.4	37.8	-0.9	6.10	2691	441	23.9	4.5
1997	15.9	3.5	7.8	16.8	3.5	3.1	27.9	1.7	4.7	3.8	0.4	25.4	-1.07	7.60	4105	540	21.8	5.0
1998	15.9	2.7	8.0	16.1	4.5	1.0	21.7	1.8	4.3	3.6	0.2	19.1	-1.53	17.7	4105	232	39.2	3.3
1999	15.1	2.3	6.7	16.5	3.5	1.1	21.5	1.6	3.6	3.3	0.2	16.6	-1.54	18.4	3716	202	37.1	3.1
2000	15.7	2.5	7.9	17.1	4.3	1.1	21.2	1.8	4.5	4.0	0.2	18.3	-1.49	16.2	3976	245	35.0	3.4
2001	15.7	2.2	7.3	16.7	3.7	0.9	18.0	2.3	3.4	2.9	0.4	15.5	-1.63	20.9	3997	191	37.8	3.1
2022	15.6	2.8	8.0	16.5	4.3	1.0	20.2	2.1	3.8	3.2	0.2	17.4	-1.57	21.9	3936	180	35.2	3.0
2003	15.7	2.5	8.2	17.2	3.7	1.2	18.8	2.2	3.2	3.1	0.2	16.6	-1.54	22.9	3992	174	39.2	3.0
2004	16.2	2.5	8.1	16.6	3.2	1.6	19.5	2.7	2.7	3.6	0.2	17.9	-1.44	18.4	4282	233	32.2	3.3
2005	14.9	2.8	8.1	17.3	3.7	1.5	22.0	1.6	3.2	4.4	0.2	17.5	-1.71	16.2	3621	224	34.2	3.2
2006	15.3	2.9	8.1	16.2	4.1	1.2	23.0	2.7	3.0	4.5	0.3	19.9	-1.61	24.2	3818	158	45.1	2.9
2007	14.4	3.0	8.4	16.7	3.8	1.2	24.3	2.2	3.2	4.3	0.3	21.5	-1.60	20.9	3383	162	48.1	2.9
2008	14.4	3.1	8.4	17.2	3.7	1.5	24.9	2.6	2.73	4.41	0.25	23.6	-1.65	21.4	3366	157	48.5	2.9
2010	14.1	3.1	8.5	17.0	3.8	1.3	25.4	2.0	2.67	4.05	0.26	24.1	-1.64	24.1	3243	135	57.3	2.7
2011	16.2	1.9	8.2	17.6	3.8	0.5	14.2	1.4	3.01	2.28	0.15	12.2	-1.94	31.4	4235	135	52.7	2.7
2012	15.9	1.3	8.1	16.9	3.1	0.5	8.6	1.3	2.67	1.93	0.11	7.4	-1.87	28.8	4102	142	29.2	2.8
2013	15.7	1.4	8.2	17.1	3.3	0.7	9.7	1.2	2.75	2.15	0.12	9.2	-1.74	24.2	3999	165	27.6	2.9
2014	15.8	1.4	8.2	16.8	3.3	0.9	9.4	1.5	2.87	1.89	0.11	9.3	-1.6	20.9	4050	194	23.4	3.1
2015	15.7	1.4	8.3	17.3	2.8	0.8	9.6	1.3	2.41	1.88	0.18	9.5	-1.7	24.1	3989	166	27.5	2.9
2016	15.6	1.5	8.2	15.2	3.3	0.7	10.4	1.8	2.69	2.01	0.13	9.9	-1.7	24.2	3968	164	26.9	2.9
2017	15.5	1.5	8.2	16.6	3.3	0.8	10.9	1.4	2.76	1.77	0.13	10.6	-1.7	24.1	3898	162	29.5	2.9
2018	15.0	1.6	8.2	17.5	3.1	1.1	12.2	1.6	4.76	3.52	0.14	10.9	-1.4	14.8	3626	245	20.6	3.4
2019	14.3	1.5	8.1	17.1	3.5	1.4	10.3	1.6	2.6	2.1	0.1	9.8	-1.48	16.4	3329	203	20.0	3.1
2020	15.3	1.5	8.2	17.3	3.1	0.8	11.1	1.3	2.6	2.0	0.1	9.4	-1.71	24.1	3793	157	29.4	2.9
2021	15.1	1.6	8.2	17.5	3.7	0.6	11.2	1.3	2.56	1.9	0.1	10.2	-1.83	28.7	3714	129	33.7	2.7
**mean**	14.5	2.8	8.1	16.9	3.8	2.0	21.9	2.0	3.3	3.8	0.2	20.8	-1.5	18.9	3563	220	34.4	3.2
**1SD**	1.3	1.4	0.4	0.5	0.6	2.0	12.3	0.7	0.8	2.0	0.1	13.2	0.3	7.4	604	96	9.2	0.5

5. Lake Zóñar hydrological balance under spring diversion and drier conditions

We evaluate the impact of climate aridification and spring diversion on the level of Lake Zóñar. The hydrological balance model used herein is described in Supplementary fig. S3 and based on the following mass balance equation:

$$L_t = L_{t-1} + I_{sup} + I_{sub} + PP_{dir} - O_{sub} – O_{sup} - EVP_{dir}$$

where:

L_t = lake level at a given time (t), where t in years, calculated from the volume-level relationship in Supplementary fig S4.

L_{t-1}= mean lake level at previous year.

I_{sup}= surface runoff from the watershed (dam^3/year).

I_{sub}= groundwater inflow (springs contribution) (dam^3/year).

PP_{dir}= direct precipitation on the lake surface (function of lake surface area at time t) (dam^3/year).

O_{sub}= groundwater outlet (dam^3/year).

O_{sup}= surface outlet to Lake Chica (dam^3/year).

EVP_{dir} = direct evaporation from the lake surface (function of lake surface area at time t) (dam^3/year).

We use annual mean parameters (i.e., runoff, spring discharge, etc.) of the modern lake and the watershed calculated by Moral-Martos et al. (2008), as estimated from meteorological and hydrological parameter of the Lake Zóñar meteorological station, and direct measurements of the lake level and spring discharge over the period 1985-1996 (12 years) (Supplementary table S4). The initial lake level (t=0) is set to the mean value over the same period (14.5 m), corresponding to a lake volume of 3411 dam^3, derived from the volume-depth relationship described in Supplementary fig. S4.

Supplementary table S4. Hydrological parameters of Lake Zóñar and its watershed obtained by Moral-Martos et al. (2008) for the period 1985-1996.

Hydrological parameters	Inflows/Outflows (dam^3/yr)
I_{sup}	807
I_{sub}	323
EVP_{dir}	787
PP_{dir}	222
O_{sub}	242

Our model considers changes in the lake surface by linking the input by direct precipitation (PP_{dir}) and the output by evaporation (EVP_{dir}) to the changes in the lake water surface. We assume that the O_{sub} does not change with time and the O_{sup} is 0, since no surface outflow toward Lake Chica is expected when the lake level is below 16 m (Moral-Martos et al., 2008).

We conduct two experiments to simulate the effect of artificial springs diversion on the lake level, where the I_{sub} (i.e., spring inlet) is reduced by 50% and by 100% of its modern value (323 dam^3/yr), while other parameters are held constant. In a second scenario, we simulated the effect of climate aridification, without modifying artificially the springs contributions. For this, all the input parameters, including the I_{sup}, I_{sub} and PP_{dir} are reduced by a percentage of their modern values, while the EVP_{dir} is increased by the same percentage. Finally, we explore the combined effect of climate aridification and anthropic spring diversion by reducing the input parameters I_{sup} and PP_{dir} and increasing EVP_{dir}, while the I_{sub} is set to a percentage of modern.

6. Isotope Model Parametrization

6.1. Temperature

The mean annual air temperature in the Lake Zóñar region was 17.2±1°C in the period from 1987 to 2021 (Supplementary fig. 2). Here we assume that, at a paleoclimate scale, the mean lake water temperature changes synchronous with the

mean air temperature in the lake setting. This means that the normalized relative humidity (RHn) is equal to the actual atmospheric relative humidity. We use water and air temperatures of 17.2°C to model the isotopic composition of modern lake water. The Iberian Roman Humid Period (IRHP) temperature is assumed to have been slightly higher than at present (18±1ºC) as suggested by SST reconstructions in the Mediterranean region (Cisneros et al., 2016). Note that the model is relatively insensitive to small temperature variations (Gázquez et al., 2018).

6.2. Isotopic composition of the freshwater input

The $\delta^{18}O$, δD and d-excess values of the freshwater input were set using the mean values of the Zóñar and Escobar spring feeding Lake Zóñar for the 2003-2005 period ($\delta^{18}O$=-5‰ and δD=-33‰, Martín-Puertas et al., 2018). These values agree with that obtained for the Zóñar spring in July 2022 ($\delta^{18}O$=-5‰ and δD=-33‰). No $\delta^{17}O$ data is available for samples from 2003-2005. Therefore, the $^{17}O_{excess}$ value was constrained by measurements of the Zóñar spring in July 2022 (22±10 per meg). We further assume that the long-term average isotope composition of rainwater is like that of the springs. High-resolution reconstructions of the isotopic of paleo-rainfall in this region are not available. However, a study of global paleo-rainfall isotope compositions suggests that in this region rainfall had similar or slightly higher $\delta^{18}O$ (0.5‰) during the last glacial maximum (LGM) compared to the modern conditions (Jasechko et al., 2015). The climatic differences between the LGM and present are expected to be less than those between the IRHP and present. Indeed, recent investigation of hydrogen stable isotopes in plant biomarkers in lake sediments of Sierra Nevada (less than 150 km from Lake Zóñar) suggest that the mean δD of rainwater between 2500 and 1500 cal yr. BP changed less than 5‰ (Toney et al., 2020). According to the global $\delta^{18}O$-δD relationship in meteoric waters (Craig et al., 1961; Dansgaard, 1964), a change of 5‰ in δD is associated to a maximum change in $\delta^{18}O$ of 0.7‰, that cannot explain the isotopic difference observed between the paleo-lake water and the modern lake water. We assume that the isotopic composition of the inlets to the lake (rainfall and springs) during the IRHP was like modern values. As noted by Gázquez et al. (2018), when keeping other model input

parameters constant, a 0.5‰ change in $\delta^{18}O$ of the freshwater member results in relatively small uncertainties of 1.5 to 2.3% in the RH estimate.

6.3. Isotopic composition of the atmospheric vapor

In the absence of direct measurements, the isotopic composition of atmospheric water vapor is often approximated by assuming equilibrium with local meteoric water. However, the IMB model does not reproduce the measured $\delta^{18}O$, $\delta^{17}O$ and δD values for any reasonable set of input parameters when full equilibrium is assumed. Gibson et al. (2016) provides a theoretical framework on how atmospheric vapor typically deviates from equilibrium with local meteoric water. These authors show that the isotope composition of atmospheric water vapor is a primary control of the slope of the evaporation trend in δD vs $\delta^{18}O$ space. This correlation is used here to infer the isotopic composition of the vapor to reasonable values. The modern-day evaporation slope observed for the Lake Zóñar region is 4, consistent with 70-75% equilibrium conditions. Slopes around 4-5 and 70-75% precipitation-vapor equilibrium are typical of temperate regions (Gibson et al. 2016). We use 70±5% equilibrium conditions between the rainfall and atmospheric water vapor during the IRHP and at present (i.e., $\delta^{18}O_v$=-11.43‰).

Supplementary table S5. Constraints on model input parameters. The isotopic composition of the lake water was modeled based on the $\delta^{18}O$, δD and $^{17}O_{excess}$ parameters, for the modern conditions and the expected conditions during the driest phases of the Iberian Roman Humid Period.

Parameters	Modern	±1SD	IRHP	±1SD
$\delta^{18}O$inflow (‰)	-5.0	0.1	-5.0	0.1
δDinflow (‰)	-33	1	-33	1
^{17}O-excess inflow (per meg)	20	5	20	5
Temperature (ºC)	17.2	1	18	1
Relative Humidity (%)	70	5	60	5
At vapor/feshwater eq (%)	70	5	70	5
Wind effect (0-1)	0.55	0.05	0.55	0.05
Evaporation/Inflow (E/I)	0.7	0.2	1.5	0.2
Salinity (g/l)	1	1	20	5

6.4. Wind Effect

It is known that the proportion of $^*\alpha_{diff}$ may be suppressed by turbulent flow induced by wind (e.g., Uemura et al., 2010). Hence wind could significantly alter isotope mass balance, especially for d-excess and ^{17}O-excess. In the IMB model wind can be accounted for by replacing $^*\alpha_{diff}$ by $(^*\alpha_{diff})^w$, where the exponent 'w' is set between 0 (pure turbulence) and 1 (no wind) (Dongmann et al., 1974; Uemura et al., 2010; Haese et al., 2013). Previous studies on the isotopic composition of evaporated waters used similar "w" values, ranging from 0.5 to 0.85 (Gázquez et al., 2018; Surma et al., 2018; Voigt et al., 2021). Gonfiantini et al. (2018) suggest that a "w" value of 0.5 should be used for evaporation environments. We use a "w" value of 0.55±0.05 to model the modern and the IRHP conditions of Lake Zóñar. This value produces the best fit of the analytical data to the model, while using other parameters produces unreasonable or no result for the modern data.

6.5. Salinity effect

The total dissolved salts (TDS) in the modern Lake Zóñar is 0.5-1.0 g/L. Significant salinity effects on the isotope composition of the lake water at such low TDS concentrations are not expected (Sofer and Gat, 1975; Criss 1999). The salt effect is insignificant on $^{17}O_{excess}$, although can impact δ^{18}O, δ^{17}O and δD parameters (Barkan et al., 2022). We assume that before the lake low stands during the IRHP the hydrochemical characteristics of the lake were similar to modern conditions. Then, we calculated the percentage of evaporative water loss required to reach gypsum saturation using Phreeq® software. Further, salinity (g/L) and conductivity (mS/cm) of the gypsum saturated solution were determined (see main text). We found that the salinity of the saturated gypsum solution would be 18.2 g/L at gypsum saturation when using the mean hydrochemical composition of lake water for selected years between 1991 and 2021. Based on these calculations, we use a mean salinity value of 20±5 g/l to model the isotopic composition of the lake during the IRHP. Note that the gypsum-water fractionation factors are largely unaffected by temperature in the range from 10 ^{o}C to 35 ^{o}C and change insignificantly with salinities below 100 g/L (Gázquez et al., 2017; Liu et al., 2018).

6.6 The evaporation-to-inflow ratio

Hydrological studies of the modern Lake Zóñar suggest that most of the water is lost from the system via evaporation (75%) and little water is lost by discharge to the aquifer via infiltration through the lake sediments and occasional overflow to Lake Chica (25%) (Moral et al., 2008). According to this study (and assuming relatively stable lake volume on inter-annual scale), the evaporation-to-inflow ratio of Lake Zóñar is thus ~0.7. For modelling the modern isotopic composition of the lake, we use an evaporation-to-inflow ratio of 0.7±0.2. For the IRHP, the IMB model does not reproduce the measured $\delta^{18}O$, $\delta^{17}O$ and δD ratios for any reasonable set of input parameters when an evaporation-to-inflow ratio ≤1 is considered. In contrast, a higher evaporation-to-inflow ratio of 1.4-1.6 are needed to reproduce the isotopic values of the paleo-lake water. This suggests that gypsum formed when the lake was in desiccating conditions, i.e., evaporation was higher than the water input to the lake. We use an evaporation-to-inflow ratio of 1.5±0.2 to model the isotopic composition of the paleo-lake water.

6.7. Relative humidity

The only fit of the model to the modern isotopic composition of Lake Zóñar is obtained for RH of 70±5% (h=0.7±0.05). Note that this value is similar to the modern mean RH in the surroundings of the lake (70±5% at Zóñar station in the period 1987-2008). This RH value is slightly higher and shows less inter-annual variability than at other nearby meteorological stations (e.g., 62±18%, at the Santaella station, 15 km away from Zóñar, in the period 2000-2020; 58±19% at the Cabra station, 20 km away from Zóñar, in the period 2003-2022 (RIA, 2022)). At present, the higher RH near the lake may indicate that the water evaporation contributes to increase the atmospheric moisture in the surroundings of the lake compared to locations more distant from the lake. This effect of humidity buildup has been observed in other lakes elsewhere (Jasechko et al., 2014). The best fit for paleo-lake water is obtained for RH of 60±5%. This RH is ~10% lower than present-day values at Lake Zóñar meteorological station, but in line with the modern RH recorded by the

meteorological stations at Cabra and Santaella. This may indicate that the regional RH in this area was lower than present during the gypsum precipitation periods of the IRHP. More likely, the reduction of water volume implied by evaporation-to-inflow ratio > 1, lead to a reduction in the water vapor derived from lake evaporation and lower impact on local relative humidity in the lake surroundings.

Sample	Age (cal. yr BP)/date	RH (%)	1SD	E/I	1SD
ZONAR 1B 1K-5 57 cm	1890	59.3	2.7	1.43	0.20
ZONAR 1B 1K-5 70 cm	2050	57.9	3.8	1.42	0.17
ZONAR 1B 1K-5 76 cm	2120	55.4	2.1	1.59	0.15
Modern surface water	28/07/2020	69.9	3.0	0.58	0.06
Modern surface water	19/03/2021	71.3	3.3	0.63	0.10
Modern surface water	30/06/2021	66.8	3.7	0.81	0.10
Modern surface water	2/07/2022	65.3	2.4	1.01	0.09

Supplementary table S6. Results of isotope modelling of pale-lake waters, reconstructed from stable isotopes in gypsum hydration water and modern surface lake water (see Supplementary table S1 for isotope input data and Supplementary table S5 for model parameters).

7.7 Archaeological observations

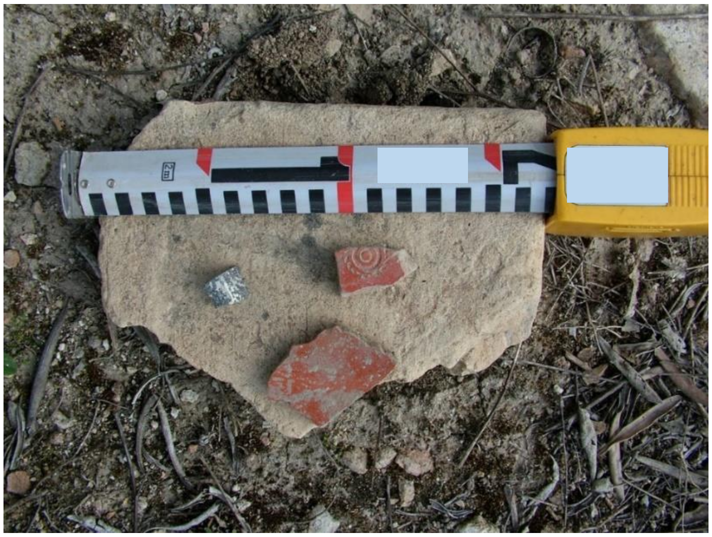

Supplementary fig. S8. Ancient Roman pottery (*terra sigillata*) and a *tesserae* (mosaic piece) on a *tegula* fragment collected in the area of the Zóñar Roman *villae* (Fig. 1 in the main text). Based in ceramic typology (Fernandez Ochoa et al., 2015, 2017 and 2019) and previous studies of Roman *villae* in this region (e.g., Hidalgo Prieto, 2017) these materials can be attributed to a Roman *villa* from the latest 1st century BCE to ~4th/5th century CE in the Baetica province (photograph taken by Dr. Dolores Ruíz-Lara).

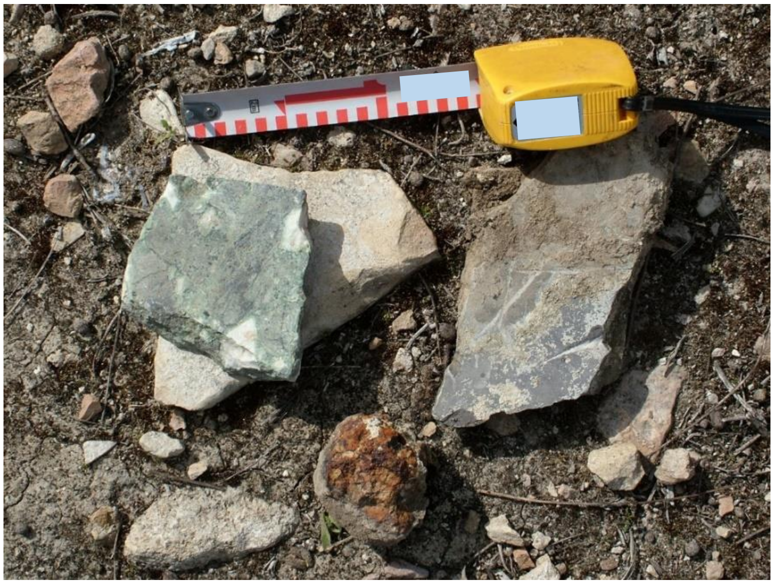

Supplementary fig. S9. Characteristic fragments of Roman marble used as pavement (*opus sectile*) together with remains of metallic elements collected in the area of the Zóñar Roman *villae* (Fig. 1 in the main text) (photograph taken by Dr. Dolores Ruíz-Lara).

References

Barkan, E., Fishman, E., Affek, H.P. The effect of salinity on water $^{17}O/^{16}O$ ratios in brines. Earth Plant. Sci. Lett. **595,** 117761 (2022).

Cisneros, M., et al. Sea surface temperature variability in the central-western Mediterranean Sea during the last 2700 years: a multi-proxy and multi-record approach. *Clim. Past* **12**, 849-869 (2016).

Craig, H. Isotopic variations in meteoric waters. Science **133**, 1702–1703. doi: 10.1126/science.133.3465.1702 (1961).

Criss, R.E., Principles of Stable Isotope Distribution. Oxford Univ. Press, Oxford, U.K. (1999).

Dansgaard, W. Stable isotopes in precipitation. Tellus **16**, 436–468. doi: 10.1111/j.2153-3490.1964.tb00181.x (1964).

Dongmann, G. et al. On the enrichment of $H_2^{18}O$ in the leaves of transpiring plants. *Radiat. Environ. Biophys.* **11,** 41–52 (1974).

Gázquez F., Evans N.P. and Hodell D.A. Precise and accurate isotope fractionation factors ($\alpha^{17}O$, $\alpha^{18}O$ and αD) for water and $CaSO_4{\cdot}2H_2O$ (gypsum). *Geochim. Cosmochim. Acta* **198,** 259–270 (2017).

Gázquez, F., et al. Triple oxygen and hydrogen isotopes of gypsum hydration water for quantitative paleohumidity reconstruction. *Earth Planet. Sci. Lett.* **481,** 177–188 (2018).

Gibson, J.J., Birks, S.J., Yi, Y. Stable isotope mass balance of lakes: a contemporary perspective. Quat. *Sci. Rev.* **131**, 316–328 (2016).

Gonfiantini, R., et al. A unified Craig-Gordon isotope model of stable hydrogen and oxygen isotope fractionation during fresh or saltwater evaporation. *Geochim. Cosmochim. Acta* **235**, 224–236 (2018).

Haese, B., Werner, M., Lohmann, G. Stable water isotopes in the coupled atmosphere–land surface model ECHAM5-JSBACH. *Geosci. Model Dev.* **6,** 1463–1480 (2013).

Jasechko, S., Gibson, J.J., Edwards, T.W.D., Stable isotope mass balance of the North American Great Lakes. *J. Gt. Lakes. Res*. **40**, 336-346 (2014).

Jasechko, S. et al., Late-Glacial to late-Holocene shifts in global precipitation δ18O. Clim. Past. **11**, 1375–1393 (2015).

Liu, T. et al. Prediction of Equilibrium Isotopic Fractionation of the Gypsum/Bassanite/Water System using First-Principles Calculations. Geochim. Cosmochim. Acta. **244**, 1-11 (2018).

Martín-Puertas, C. et al. The Iberian–Roman Humid Period (2600–1600 cal yr BP) in the Zóñar Lake varve record (Andalucía, southern Spain). *Quat. Res*. **71**, 108–120 (2009).

Moral-Martos, et al. Definición del contexto hidrológico de humedales de la campiña andaluza central. Memoría técnica. Confederación hidrográfica del Guadalquivir. (2008). Junta de Andalucía public report (https://www.chguadalquivir.es/documents/10182/52021/Humedales_Campina_Andaluza_I.pdf/62460c86-aa37-4630-91a9-b0a7e55b946a)

RIA, 2022. Red de Información agroclimática de Andalucía (https://www.juntadeandalucia.es/agriculturaypesca/ifapa/riaweb/web/).

Sánchez de la Orden, M., Fernández-Delgado, C. & Sánchez Polaina, F. Nuevos datos acerca de la morfometría y batimetría de la laguna de Zóñar (Aguilar de la Frontera, Córdoba). Oxyura.VI, 1, 73-77 (1992).

Sofer, Z., Gat, J.R. The isotope composition of evaporating brines: effect of the isotopic activity ratio in saline solutions. *Earth Planet. Sci. Lett.*26, 179–186. (1975).

Surma, J. et al. The evolution of ^{17}O-excess in surface water of the arid environment during recharge and evaporation. *Sci. Rep.* **4972** (2018).

Toney, J.L. et al. New insights into Holocene hydrology and temperature from lipid biomarkers in western Mediterranean alpine wetlands. *Quat. Sci. Rev.* **240**, 106395 (2020).

Uemera, R. et al. Triple isotope composition of oxygen in atmospheric water vapor. *Geophys. Res. Lett.* **37**, L04402 (2010).

Voigt, C. et al., Triple oxygen isotope systematics of evaporation and mixing processes in a dynamic desert lake system. *Hydrol. Earth Syst. Sci.* **25**, 1211–1228 (2021).
